# Things Are Getting Hairy: Enterobacteria Bacteriophage vB_PcaM_CBB

**DOI:** 10.3389/fmicb.2017.00044

**Published:** 2017-01-24

**Authors:** Colin Buttimer, Hanne Hendrix, Hugo Oliveira, Aidan Casey, Horst Neve, Olivia McAuliffe, R. Paul Ross, Colin Hill, Jean-Paul Noben, Jim O'Mahony, Rob Lavigne, Aidan Coffey

**Affiliations:** ^1^Department of Biological Sciences, Cork Institute of TechnologyCork, Ireland; ^2^Laboratory of Gene Technology, KU LeuvenLeuven, Belgium; ^3^Laboratório de Investigação em Biofilmes Rosário Oliveira, Centre of Biological Engineering, University of MinhoBraga, Portugal; ^4^Teagasc Food Research Centre, Moorepark Fermoy, Co.Cork, Ireland; ^5^Department of Microbiology and Biotechnology, Max Rubner-InstitutKiel, Germany; ^6^APC Microbiome Institute and School of Microbiology, University CollegeCork, Ireland; ^7^Biomedical Research Institute and Transnational University Limburg, Hasselt UniversityDiepenbeek, Belgium

**Keywords:** bacteriophages, genome, bioinformatics, mass spectrometry, Jumbo bacteriophage, PFGE analysis, host range, transmission electron microscopy

## Abstract

Enterobacteria phage vB_PcaM_CBB is a “jumbo” phage belonging to the family *Myoviridae*. It possesses highly atypical whisker-like structures along the length of its contractile tail. It has a broad host range with the capability of infecting species of the genera *Erwinia, Pectobacterium*, and *Cronobacter*. With a genome of 355,922 bp, excluding a predicted terminal repeat of 22,456 bp, phage CBB is the third largest phage sequenced to date. Its genome was predicted to encode 554 ORFs with 33 tRNAs. Based on prediction and proteome analysis of the virions, 29% of its predicted ORFs could be functionally assigned. Protein comparison shows that CBB shares between 33–38% of its proteins with Cronobacter phage GAP32, coliphages PBECO4 and 121Q as well as *Klebsiella* phage vB_KleM_Rak2. This work presents a detailed and comparative analysis of vB_PcaM_CBB of a highly atypical jumbo myoviridae phage, contributing to a better understanding of phage diversity and biology.

## Introduction

Bacteriophages (phages) the viruses of bacteria are the most abundant biological entities in the biosphere with an estimated number of 10^31^ (Whitman et al., 1998; Hendrix, 2002). The order *Caudovirales* (the tailed phages) make up the greatest majority of the phages types that have been studied and within this order are the phage families of *Myoviridae, Siphovirdae*, and *Podoviridae* (Ackermann, 2001). *Myoviridae* have the most sophisticated virion design, possessing a tail capable of contracting on infection and generally having the largest genomes when compared to the other families (Hatfull, 2008). However, only a small number of the known *Myoviridae* phages have genomes greater than 200 kbp; and these are often referred to as “giant” or “jumbo” phages (Hendrix, 2009). The largest of these isolated to date are *Bacillus megaterium* phage G (498 kbp, accession no. JN638751.1), *Cronobacter* phage GAP32 (358 kbp, accession no. NC_019401), *Escherichia* phage PBECO4 (348 kbp, accession no. NC_027364) and *Klebsiella* phage Rak2 (345 kbp, accession no. NC_019526). The former three phages, GAP32, PBECO4, and Rak2 are recent discoveries, and their genomes have only been presented within the last 3 years (Kim et al., 2013; Šimolūnas et al., 2013; Abbasifar et al., 2014). These phages share a number of protein homologs with the T4-like phages, but they lack a number of universal core proteins found among the *Myoviridae* subfamily of *Tevenvirinae*. Due to the lack of sequence identity, the lack of an even distribution of homologs among their genomes with the T4-like phages and the possession of their own species-specific proteins, it has been proposed that these phages should be placed within a new subfamily (Abbasifar et al., 2014). More recently, there have been two additional phages that share homology to this subfamily (termed the Rak2-like phages from here onwards in this article) these are *Escherichia* phage 121Q (348 kbp, accession no. NC_025447.1) and *Klebsiella* phage K61-1 (346 kbp, accession no. AB897757).

This article presents morphological, genomic and structural proteomic findings on another newly isolated Rak2-like phage, Enterobacteria phage vB_PcaM_CBB. This is a broad host range jumbo phage which possesses highly atypical whisker-like structures along its contractile tail surface, a feature which has not been described in any of the other Rak2-like phages published to date.

## Methods

### Bacterial strains, phage, and cultivation conditions

Phage CBB was isolated and propagated on *Pectobacterium carotovorum subsp carotovorum* strain CBBL19-1-37. Bacterial strains used for the host range study are listed in Supplementary S1, Table S1. Crystal Violet Pectate selective agar (Hélias et al., 2012) was used to isolate *Pectobacterium* strains from potatoes presenting symptoms of blackleg which were obtained from farms in Co. Cork, Ireland. Bacterial identification was achieved using biochemical tests as well as genus- and species-specific PCRs (Darrasse et al., 1994; De Boer and Ward, 1995; Kang et al., 2003). To grow bacterial stains and propagate the phage, Lysogeny broth (LB), LB agar (1.5% w/v agar), and LB overlays (0.2 w/v agarose) were used (Sigma- Aldrich, St. Louis, Missouri, United States). Incubation temperatures of 25°C were used for all cultures used in this study.

### Phage techniques

Phage enrichment and isolation was conducted as described by Van Twest and Kropinski (2009). The phage double overlay assay was conducted using overlays containing 0.2% agarose for morphologically large phage as described by Serwer et al. (2007). Stocks of phage were produced according to the plate lysis method described by Sambrook and Russell (2001a). To determine phage titre, a 10-fold dilution series of phage stock was tested with agarose overlay as described previously Sambrook and Russell (2001b). Host range assays of phage CBB were performed by spot assay as described by Mazzocco et al. (2009). Isopycnic centrifugation though CsCl gradients was performed as described by Sambrook and Russell (2001c) with a number of modifications. A high titre phage lysate (>1 × 10^9^ plaque forming units (PFU)/mL), was precipitated using polyethylene glycol (15% w/v PEG8000, 1 M NaCl) at 4°C overnight and centrifuged, after which the pellet was resuspended in TMN buffer (10 mM Tris-HCl pH7.4, 10 mM MgSO_4_.7H_2_O, 0.5 M NaCl) followed by a chloroform phase separation step (1:1) to remove debris. The resulting phage preparation was placed onto a CsCl step gradient composed of 1.3, 1.5, and 1.7 g/ml layers and spun in a 100 Ti rotor (Beckman Coulter) at 200,480 *g* for 3 h at 4°C. Resulting phage bands were collected and subjected to dialysis with two changes of Tris-HCl buffer (10 mM, pH7.5) at 4°C.

### Transmission electron microscopy

Phages were negatively stained on freshly prepared carbon films with 2% (w/v) uranyl acetate. Micrographs were taken using a Tecnai 10 transmission electron microscope (FEI Thermo Fisher, Eindhoven, the Netherlands) at an acceleration voltage of 80 kV with a MegaView G2 CCD-camera (emsis, Muenster, Germany).

### DNA isolation and sequencing

DNA extraction was performed as previously described (Pickard, 2009). CsCl purified phage particles were treated with DNase and RNase, followed by treatment with 10% SDS and proteinase K followed by DNA extraction with phenol:chloroform:isoamyl alcohol (25:24:1 v/v) and chloroform: isoamyl alcohol (24:1 v/v). DNA quality and quantity were estimated using both a nanodrop (NanoDrop, ND-1000) and by visualization after agarose gel electrophoresis. Phage DNA was sequenced with a high throughput Illumina MiSeq System outsourced at Nucleomics Core (VIB, Belgium). Libraries were processed with a custom NEBNext® Ultra™ DNA Kit to generate 500-bp fragments with individual barcodes. The quality of each library preparation was controlled using an Agilent Bioanalyzer and Qubit measurements, before being pooled together with a non-homologous genome and sequenced with 2 × 150 bp paired-end reads. Demultiplex, quality controlled (above Q30) and trimmed reads were *de novo* assembled using CLC Bio Genomics Workbench v7.0 (Aarhus, Denmark) into a single contig. Contigs were resolved with 1,044,532 reads and with an average coverage of 401x for phage CBB.

### Pulsed field gel electrophoresis

Pulsed field gel electrophoresis (PFGE) was conducted as described by Lingohr et al. (2009). A CsCl purified phage suspension was mixed with an equal volume of low-melting point agarose (Bio-Rad Laboratories, Hercules, USA). Prepared plugs were placed in lysis buffer (50 mM Tris, 50 mM EDTA, 1% SDS) and digested with proteinase K for 2 h at 54°C. Plugs were then washed twice with TE buffer (10 mM Tris, 1 mM EDTA [pH8.0]) and subjected to electrophoresis on a 1% agarose gel using Bio-Rad CHEF-DR® II PFGE apparatus (Bio-Rad Laboratories, Hercules, USA) at 6 V/cm (200 V) with 60–120 switch time ramp for 24 h. Yeast chromosome PFGE markers (Bio-Rad Laboratories, Hercules, USA) were used to allow estimation of phage genome size.

### Bioinformatic analysis

The encoding potential of open reading frames (ORFs) was predicted with Glimmer (http://www.ncbi.nlm.nih.gov/genomes/MICROBES/glimmer_3.cgi; Delcher et al., 1999) and GenemarkS (http://exon.gatech.edu/genemark/genemarks.cgi; Besemer et al., 2001). Possible functions of predicted proteins were predicted with BLASTP (http://blast.ncbi.nlm.nih.gov/Blast.cgi?PAGE=Proteins), Pfam (http://pfam.xfam.org/search#tabview=tab1; Finn et al., 2015), HHpred (http://toolkit.tuebingen.mpg.de/hhpred; Söding et al., 2005) and InterPro scan (http://www.ebi.ac.uk/interpro/search/sequence-search; Mitchell et al., 2014). Molecular weights of predicted proteins were estimated using the batch protein molecular weight determination of the sequence manipulation suite (http://www.bioinformatics.org/sms2/protein_mw.html). Detection of proteins with possible transmembrane domains and the lipoprotein cleavage signal was explored with TMHMM v.2 (http://www.cbs.dtu.dk/services/TMHMM/) and LipoP v.1 (http://www.cbs.dtu.dk/services/LipoP/), respectively (Käll et al., 2004; Rahman et al., 2008). Transfer RNA genes were predicted with tRNAscan-SE (http://lowelab.ucsc.edu/tRNAscan-SE/; Lowe and Eddy, 1997) and ARAGORN (http://130.235.46.10/ARAGORN/; Laslett and Canback, 2004). Potential promoters were predicted by using extractUpStreamDNA (https://github.com/ajvilleg/extractUpStreamDNA) to extract 100 bp of DNA sequence upstream of each gene and submitting all these sequences to MEME (Multiple Em for Motif Elicitation) (http://meme-suite.org/tools/meme; Bailey et al., 2009). Rho-independent terminators were located with ARNOLD (http://rna.igmors.u-psud.fr/toolbox/arnold/; Naville et al., 2011) with predictions being verified using Mfold Quikfold using RNA energy rules 3.0 (http://unafold.rna.albany.edu/?q=DINAMelt/Quickfold; Zuker, 2003). Codon usage of CBB was analyzed using University of Georgia's amino acid and codon usage statistics services (http://www.cmbl.uga.edu/software/codon_usage.html).

Coregenes (Turner et al., 2013) was used for total proteome comparisons between phages with the BLASTP threshold set at 75% and the terminal repeat ORFs, CBB_555 to CBB_605, of phage CBB being excluded. Genome comparison of the Rak2-like phages was visualized using Easyfig (Sullivan et al., 2011) with comparison of genome sequences facilitated by TBLASTX (genomes were orientated such that the largest ORF's start codon was set to the first position to allow improved visual understanding as the starting point of all Genbank sequences was not uniform, with the terminal repeat of CBB also being excluded).

Phylogenetic analysis was conducted using MEGA version 7 (Kumar et al., 2016). Analysis using the portal vertex protein of phage CBB was conducted as described previously (Brewer et al., 2014). The full length protein was used in the BLASTP search to find phages with homologous portal proteins, taking a conserved internal region from 100 different portal vertex protein from different phages and aligned with MUSCLE. The resulting alignment was then used to create a phylogenetic tree based on the Maximum likelihood method (Jones et al., 1992) with 100 performed bootstraps.

### Phage CBB virion ESI-MS/MS proteome analysis

Phage capsid proteins were extracted from high titre CsCl purified phage (>1 × 10^9^ PFU/mL) using chloroform:methanol extraction (1:1:0.75, v/v/v). The resulting protein pellet was resuspended in loading buffer (1% SDS, 6% sucrose, 100 mM dithiothreitol, 10 mM Tris pH 6.8, 0.0625% w/v bromophenol blue) and heated to 95°C for 5 min to resuspend the pellet. This was subsequently loaded onto a 12% SDS-PAGE gel after which gel electrophoresis was conducted. The resulting gel was then stained using Gelcode™ Blue Safe Protein Stain (Thermo Scientific, Waltham, Massachusetts, United States) to visualize virion proteins. Gel fragments were extracted and subjected to trypsinization which were analyzed using tandem electrospray ionization-mass spectrometry (ESI-MS/MS) exactly as described previously (Van den Bossche et al., 2014).

### Accession number

The genome sequence of phage CBB has been submitted to Genbank under accession number KU574722.

## Results and discussion

### Growth parameters, morphology, and host range

*Enterobacteria* bacteriophage vB_PcaM_CBB was isolated from activated sludge from a waste water treatment plant in Little island, Co. Cork, Ireland. The bacterial host used was *P. carotovorum* sbp. *carovotorum* strain CBBL19-37-1 which had previously been isolated from blackleg-infected potato crop from Co. Cork. When the phage was plated and examined by plaque assay utilizing a 0.4% agar overlay, pinpoint sized plaques were observed with inconsistent formation across replicate experiments. This problem was overcome by using a 0.2% agarose overlay as described by Serwer et al. (2007). The host range of the phage was examined using a number of bacterial genera and species within the family *Enterobacteriacea* which showed it was capable of forming plaques on strains of *P. carotovorum, Pectobacterium atrosepticum, Erwinia mallotivora, Cronobacter muytjensii*, and *Cronobacter malonaticus*. The phage was also found to cause lysis-from-without on *Dickeya dianthicola, Dickeya solani, Pantoea agglomerans*, and *Erwinia amylovora*, as lysis was observed on low dilutions (neat, 10^−1^) of tested phage lysate with no plaque formation observed at subsequent higher dilutions (up to 10^−8^) (see Table 1).

**Table 1:** **Host range of bacteriophage vb_PcaM_CBB on 35 strains of various members of the bacterial family of ***Enterobacteriace*** as determined by spot testing with serial phage dilutions (the experiment was done twice: on separate days)**.

**Bacteria**	**Strain**	**Sensitivity**
*Cronobacter muytjensii*	ATCC 51329 (type strain)	2
*Cronobacter malonaticus*	DPC 6531	2
*Cronobacter sakazakii*	ATCC 29004	0
*Dickeya chrysanthemi* biovar *chrysanthemi*	LMG 2804 (type strain)	0
*Dickeya dianthicola*	PD 482	0
*Dickeya dianthicola*	PD 2174	1
*Dickeya dianthicola*	GBBC 1538	1
*Dickeya solani*	sp. PRI 2222	1
*Dickeya solani*	LMG 25865	1
*Dickeya solani*	GBBC 1502	1
*Dickeya solani*	GBBC 1586	1
*Enterobacter cloacae*	NCTC 11590	0
*Enterobacter gergoviae*	NCTC 11434 (type strain)	0
*Erwinia amylovora*	LMG 2024 (type strain)	0
*Erwinia amylovora*	GBBC 403	1
*Erwinia mallotivora*	LMG 1271	2
*Pantoea agglomerans*	LMG 2660	1
*Pantoea agglomerans*	LMG 2570	0
*Pantoea stewartii*	LMG 2713	0
*Pantoea stewartii*	LMG 2714	0
*Pantoea stewartii*	LMG 2712	0
*Pectobacterium atrosepticum*	DSM 18077 (type strain)	0
*Pectobacterium atrosepticum*	DSM30186	2
*Pectobacterium atrosepticum*	CB BL5-1	1
*Pectobacterium atrosepticum*	CB BL7-1	1
*Pectobacterium atrosepticum*	CB BL11-1	1
*Pectobacterium atrosepticum*	CB BL12-2	1
*Pectobacterium atrosepticum*	CB BL13-1	1
*Pectobacterium atrosepticum*	CB BL14-1	1
*Pectobacterium atrosepticum*	CB BL15-1	1
*Pectobacterium atrosepticum*	CB BL16-1	2
*Pectobacterium carotovorum sbp. carovotorum*	DSM 30168 (type strain)	0
*Pectobacterium carotovorum sbp. carovotorum*	DSM 30169	0
*Pectobacterium carotovorum sbp. carovotorum*	DSM 30170	0
*Pectobacterium carotovorum sbp. carovotorum*	CB BL19-1-37	2

*Results were recorded as 2 (plaque formation), 1 (lysis-from-without) and 0 (no sensitivity)*.

**Figure 1:**
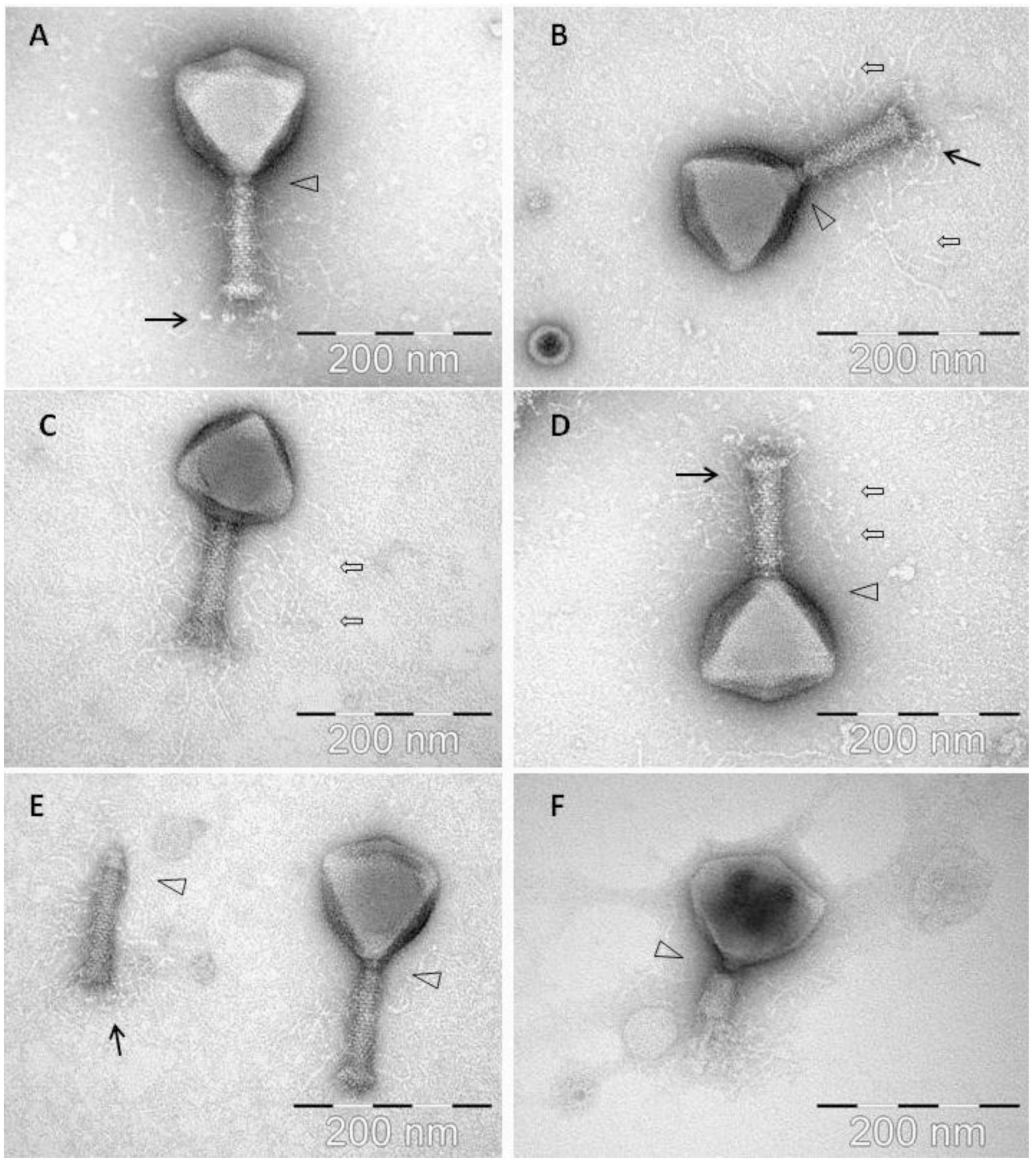
**Electron micrographs of phage CBB with black arrows indicating baseplate fibers, open arrows indicating hair-like appendages (whiskers) and triangles indicating neck passage structure. (A,B,E)** CBB virion base plate tail fibers are indicated. **(B–D)** Fully intact virions with atypical whisker-like structures on the contractile tail surface. **(E)** CBB virion contractile tail missing a capsid. **(F)** CBB virion with contracted tail.

Examination of morphology of CBB by transmission electron microscopy showed it belonged to the family of *Myoviridae* with an A1 morphotype (Ackermann, 2001), displaying large icosahedral heads presenting hexagonal and pentagonal outlines in micrographs (see Figure 1) from which estimations were made on its dimensions (see Table 2). Capsids had a height of 126.9 ± 4.9 nm and a width of 128.0 ± 6.2 nm. Tails displayed transverse striations with dimensions of 123.0 ± 2.6 × 27.1 ± 1.8 nm with a base plate with dimensions of 36.4 ± 2.3 × 14.7 ± 1.2 nm. Phage CBB also possesses a neck passage structure (see triangle in Figures 1A,B,D–F) and beneath the base plate a set of six short tail fibers (length 28.6 ± 2.7 nm) could also be visualized (see arrow in Figures 1A,B,E). Phage particles with contracted tail sheaths were only rarely found indicating a high structural stability of the phage (Figure 1F). A low number of separated phage tails without a capsid was also detected (still decorated with hairy appendices and neck passage structure (see Figure 1E). The contractile tail of CBB was found to be highly unusual as it possesses hair-like appendages (whiskers) covering its surface (see open arrows in Figures 1B–D). These hairs (length 119.3 ± 10.4 nm) are highly stable, as they did not dissociate from the phage tails during a 1½ year of storage at 4°C. Only two other phages have been reported to possess these hairy features; these being *Escherichia coli* phage PhAPEC6 (Tsones, 2014) and an unpurified phage observed in a preparation of crushed silkworm larvae referred to as X particle (Ackermann et al., 1994). Other than CBB having the whiskers, its morphology is typical of the Rak2-like phages in that it has a very large head with a comparatively short tail (Drulis-Kawa et al., 2014).

**Table 2:** **Estimations of the dimensions of Enterobacteria phage vB_Pca_CBB derived from micrographs obtained from transmission electron microscopy**.

**Head**		**Tail**		**NPS**		**Baseplate**		**Tail hairs**	**Baseplate fibers**
**Height *n* = 16**	**Width *n* = 16**	**Length *n* = 16**	**Width *n* = 16**	**Width *n* = 16**	**Height *n* = 16**	**Width *n* = 16**	**Height *n* = 16**	**Length *n* = 29**	**Length *n* = 33**
126.9 ± 4.9	128.0 ± 6.2	123.0 ± 2.6	27.1 ± 1.8	21.8 ± 1.4	7.4 ± 0.7	36.4 ± 2.3	14.7 ± 1.2	119.3 ± 10.4	28.6 ± 2.7

*Tail length: incl. NPS (collar) structure and incl. baseplate (but without bp fibers)*.

*Tail hairs: measured from flanking tail sheath surface and incl. tiny terminal globular structures*.

*Bp fibers: measured incl. terminal globular structures*.

### General genome features

**Figure 2:**
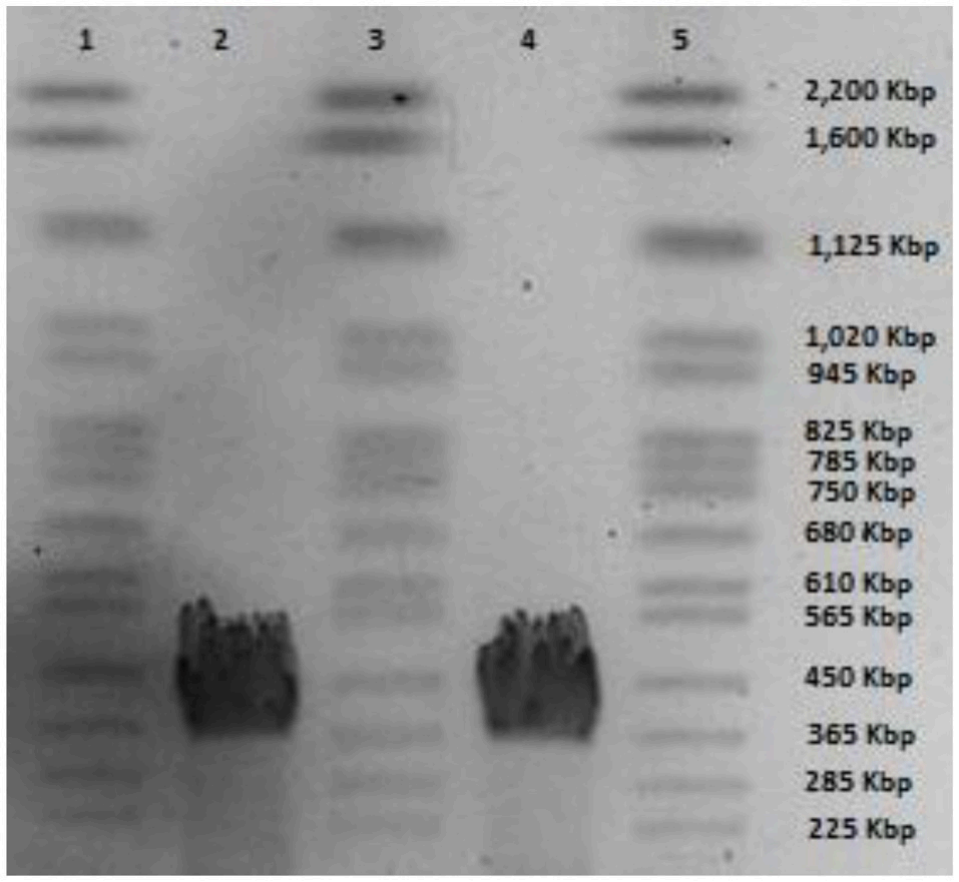
**PFGE of phage CBB genomic DNA; lanes 1, 3, and 5 yeast chromosome PFG marker (Bio-Rad Laboratories) and lane 2 and 4 genomic DNA of phage CBB**.

Phage CBB was found to possess a very large genome of 355,922 bp with predicted terminal repeats of 22,456 bp (resulting in a total genome size of 378,378 bp). The existence of terminal repeats was suggested during sequence analysis by the identification of a localized area in the genome which had twice the read depth compared with the rest of the genome. Identification of phage genome ends by this approach has been reported for other phages (Fouts et al., 2013; Li et al., 2014). The existence of the terminal repeats was verified by PFGE, which indicated that the size of genome obtained from sequencing is within the correct range (Figure 2). However, even when terminal repeats are excluded, the genome is the third largest published phage genome to date, behind *B. megaterium* phage G (497,513 bp) and *Cronobacter sakazakii* phage vB_CsaM_GAP32 (358,663 bp) (Abbasifar et al., 2014).

**Figure 3:**
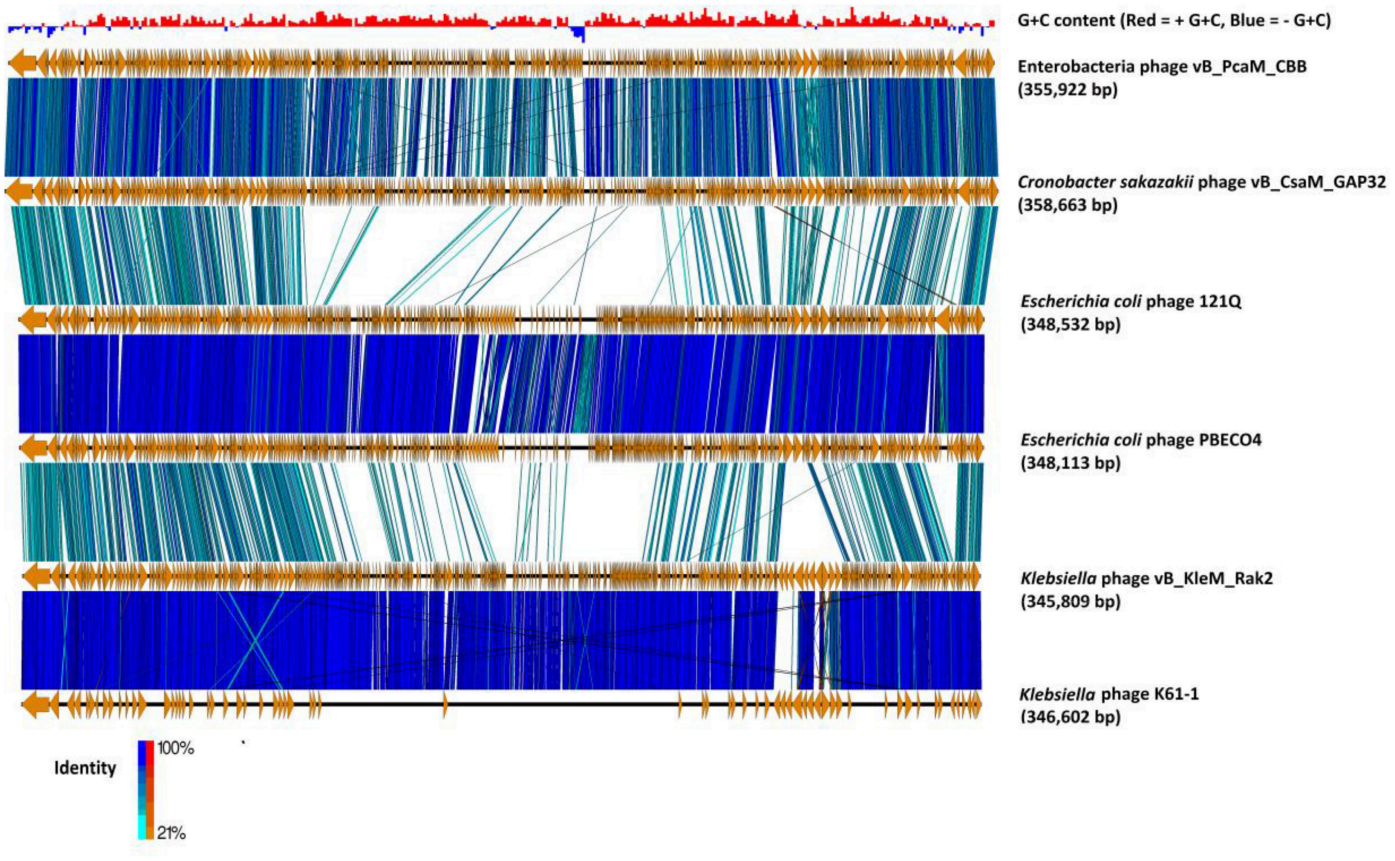
**Comparison of the genomes of phage CBB (without predicted terminal repeat region) to other potential Rak2-like phages (***Cronobacter sakazakii*** phage vB_CsaM_ GAP32, ***Escherichia coli*** phage 121Q, ***E. coli*** phage PBECO4, ***Klebsiella*** phage vB_KleM_RAK2, ***Klebsiella*** phage K64-1) using currently available annotations employing TBLASTX and visualised with Easyfig (Sullivan et al., **2011**)**. A bar chart shows the G+C skew of the CBB genome, genome maps comprise of orange arrows indicating locations of genes among the different phage genomes; and lines between genome maps indicate level of homology (blue/turquoise—genes sharing orientation, red/orange—genes in inverted orientation). To assist in the comparison between genomes the largest gene of each of the phages was positioned as the first gene for each genome.

The average G+C content of the genome of CBB is 36% with below-average skews appearing around ORFs on the minus strand (see Figure 3). The average G+C content is much lower than that typically found in its hosts species namely, *P. atrosepticum* at 50–51% (Bell et al., 2004; Nikolaichik et al., 2014), *Pectobacterium carotovorum* sbp *carotovorum* at 52.2% (Park et al., 2012) and *E. mallotivora* at 52.4% (Redzuan et al., 2014). This observation is not as unusual, as virulent phages will usually deviate from their host G+C content having a higher A+T. It has been suggested that this is due to energy and metabolism cost limitations (Rocha and Danchin, 2002). However, the G+C content is quite similar to other Rak2-like phages (35.5, 34.09, and 35.5% for GAP32, PBECO4, and Rak2, respectively).

The genome of CBB has 554 predicted Open Reading Frames (ORFs) (excluding 51 ORFs within the predicted terminal repeat), of which only 34 are encoded on the minus strand. Twenty two ORFs were found to share sequence homology with each other suggesting that these potential genes arose from duplication events (paralogs) (see Supplementary S2, Table S1). On the basis of (i) observed protein sequence homologies, (ii) protein structure homology, (iii) lipoprotein, and (iv) transmembrane analysis it was possible to predict the possible role for 162 of these proteins (see Supplementary S3, Table S1) with several others being categorized as hypothetical proteins (47), conserved hypothetical proteins (291), putative lipoproteins (4), and putative membrane proteins (7) or conserved putative membrane proteins (43). No integrase, excisionase or repressor genes were detected in the genome which suggests that this phage follows an exclusively lytic lifestyle.

It is understood that virulent phages can overcome codon utilization differences from their hosts by using encoded tRNAs, a feature often observed in large genomes (Mesyanzhinov et al., 2002). Phage CBB has a large number of predicted tRNA genes with a possible 33 being identified and concentrated within a ~51 kbp region within the genome. Thirty one of these genes appear to encode functional tRNA genes, with many of the tRNAs being for amino acids with codons which are highly utilized by the phage (see and Supplementary S2, Table S4 and Supplementary S4, Table S1).

### Phage CBB: a Rak2-like phage and its distant relationship to the tevenvirinae subfamily

Initial BLASTP searches with the predicted ORFs of phage CBB showed that it shared strong homology with the Rak2-like phages. Comparisons among these phages showed that the closest relative to CBB is *C. sakazaki* phage GAP32 (358,663 bp; 545 ORFs), with 479 homolog proteins followed by *E. coli* 121Q (348, 532; 611 ORFs) with 239 homolog proteins, *E. coli* PBECO4 (348,113 bp; 551 ORFs) with 230 homolog proteins and *Klebsiella* phage RAK2 (345,809 bp; 554 ORFs) with 230 homolog proteins. Phage CBB also shares strong homology with *Klebsiella* phage K64-1 (346,602 bp), however, at present its Genbank file contains an incomplete annotation (see Figure 3). Comparison between the proteomes of these phages shows that they share between 33 and 38% of their proteins (204 homologous proteins). These shared proteins likely represent the core genome of the Rak2-like phages, given that they appear to be resistant to sequence deviation and horizontal gene exchange due to their importance for successful phage infection.

It has been reported that the Rak2-like phages possess a number of proteins that are homologous to those found within the superfamily of T4-like phages (Šimolūnas et al., 2013; Abbasifar et al., 2014). Total protein comparisons of phages CBB, GAP32, PEBEC04, RAK2 with phage T4 (NC_000866) and phage KVP40 (NC_005083.2) show that they share 41 homologous proteins with T4 (14.75% of the total proteins of T4) and 46 homologous proteins with KVP40 (12.07% of the total proteins of KVP40) at BLASTP cut value of 75%. However, despite this correlation to the *Tevenvirinae subfamily* it is clear that the vast differences suggest these phages should be grouped separately. Nevertheless, a clear evolutionary link is present. There are 38 proteins which are considered to be the core proteins of the T4-like viruses. These proteins range in function from DNA replication, repair and recombination, auxiliary metabolism, gene expression and phage morphogenesis (Petrov et al., 2010). Examination of the CBB genome revealed that it has ORFs for proteins that appear to be homologs to these core proteins, with 21 being identified with functions involved in DNA replication and recombination, auxiliary metabolism as well as phage morphogenesis proteins with homology to those of T4 and KVP40 (reference strains for *T4likevirus* and *Schizot4virus*, respectively). However, large divergence was observed, especially with morphologically-related proteins, with some having very little homology to those of the reference strains (see Table 3). As well as T4-like core genes, there are a number of genes that have been designated at T4-like quasicore genes. These are genes whose presence will vary from phage to phage as it is believed that they are not necessary in certain genetic backgrounds (Petrov et al., 2010). In the genome of CBB it was also possible to identify 12 ORFs specifying putative homologs of these quasicore proteins (see Supplementary S2, Table S2).

**Table 3:** **Core proteins of T4-like phages identified within the CBB genome with full length sequence comparison to homologs in Enterobacter phage T4 and ***Vibrio*** phage KVP40 (reference strains of ***T4likevirus*** and ***Schizot4likevirus***, respectively) using BLASTP**.

**Function**	**T4 like core protein**	**CBB homolog**	**Accession number of T4 protein**	**Identity (%)**	***E*-value**	**Accession number of KVP4D protein**	**Identity (%)**	***E*-value**
DNA replication, repair and recombination	gp43 - DNA polymerase	CBB_263	NP_049662.1	28	4.00E-26	NP_899330.1	29	4.00E-37
	gp44- sliding clamp complex	CBB_332	NP_049665.1	30	6.00E-28	NP_899326.1	27	5.00E-25
	gp41- helicase-primase complex	CBB_291	NP_049654.1	25	1.00E-21	NP_899258.1	25	4.00E-28
	gp32 - single strand binding protein	CBB_277	NP_049854.1	29	3.00E-08	NP_899253.1	23	0.005
	gp46 - subunits of recombination nuclease	CBB_246	NP_049669.1	31	1.00E-23	NP_899322.1	30	3.00E-21
	gp47 - subunits of recombination nuclease	CBB_245	NP_049672.1	25	6.00E-17	NP_899320.1	24	8.00E-14
	UvsW protein - recombination DNA-RNA helicase	CBB_281	NP_049796.1	23	3.00E-29	NP_899623.1	27	6.00E-46
Auxiliary metabolism	NrdA - ribonucleotide reductase	CBB_303	NP_049845.1	46	0	NP_899523.1	47	0
	NrdB - ribonucleotide reductase	CBB_305	NP_049841.1	40	3.00E-105	NP_899524.1	45	9.00E-122
Phage morphogenesis	gp4 - head completion protein	CBB_208	NP_049755.1	34	1.00E-21	NP_899577.1	42	2.00E-25
	gp6 - baseplate wedge component	CBB_237	NP_049764.1	42	0.098	NP_899587.1	27	3.00E-05
	gp13 - neck protein	CBB_201	NP 049772.1	31	1.00E-06	NP 899596.1	21	3.00E-07
	gp15 - tail completion protein	CBB_233	NP_049774.1	20	3.00E-07	NP_899598.1	21	2.00E-05
	gp17 - large terminase	CBB_275	NP_049776.1	28	8.00E-29	NP_899601.1	25	2.00E-42
	gp18- tail sheath subunit	CBB_204	NP_049780.1	36	2.00E-27	NP_899602.1	39	4.00E-32
	gp20 - head portal vertex protein	CBB_252	NP_049782.1	29	2.00E-36	NP_899604.1	31	2.00E-35
	gp21- prohead core protein	CBB_255	NP 049785.1	31	2.00E-10	NP_899607.1	37	1.00E-25
	gp23 - precursor to major head protein	CBB_257	NP_049787.1	35	3.00E-36	NP_899609.1	34	8.00E-37
	gp25 - base plate wedge subunit	CBB_238	NP_049800.1	31	2.00E-10	NP_899586.1	28	1.00E-08
	gp53 - baseplate wedge component	CBB_242	NP_049756.1	38	1.00E-04	NP_899579.1	35	5.00E-07

**Figure 4:**
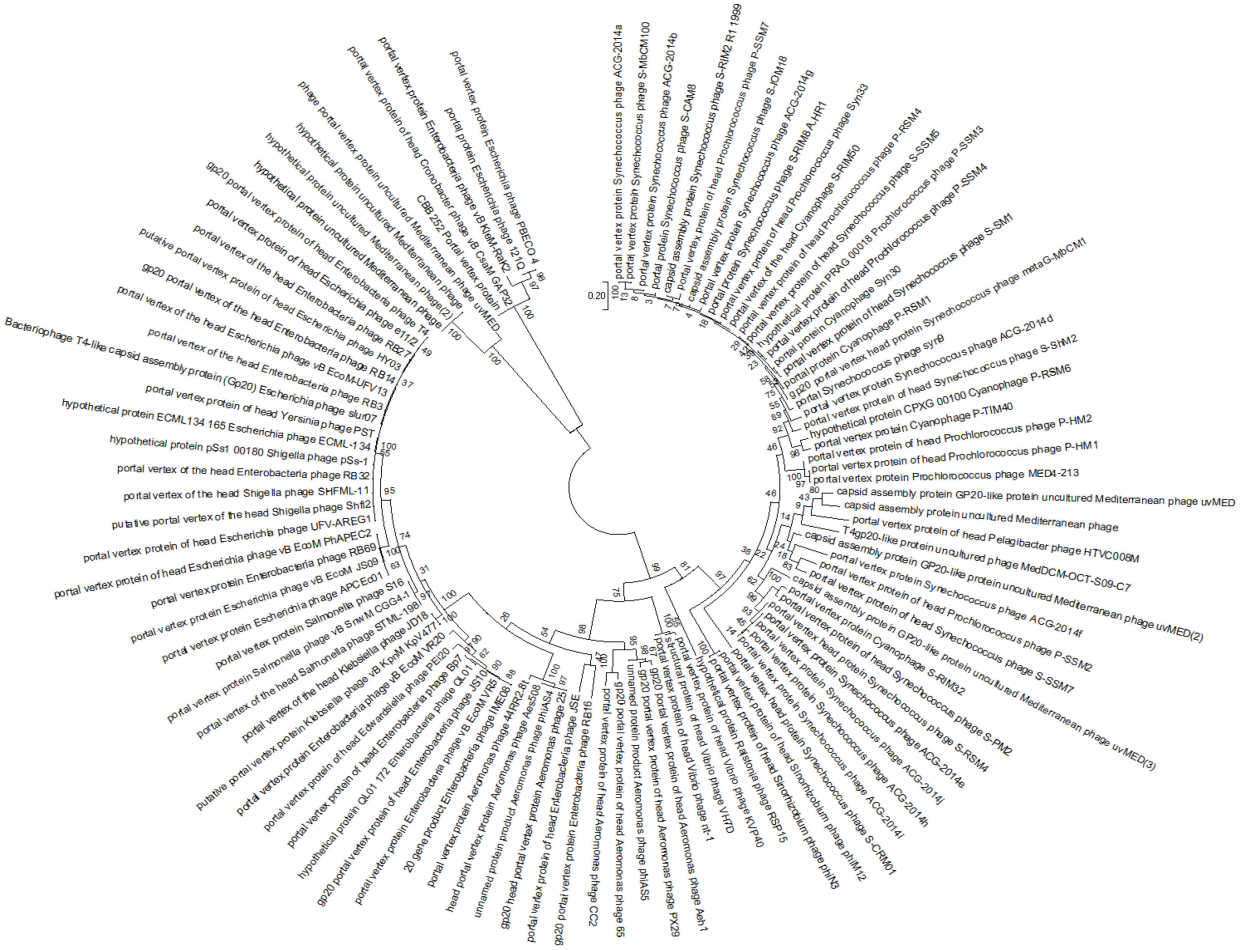
**Maximum likelihood tree created from the alignment of the conserved region of the portal vertex region of 100 homologous sequences from different T4-like phages to that of the portal vertex protein of phage CBB, found using a BLASTP search**.

To further investigate the relationship between CBB and the other Rak2-like phages and the T4-like phages, the amino acid sequence of portal vertex protein (CBB_252) of CBB was used to construct a phylogenetic tree using the top BLASTP hits against CBB_252 (see Supplementary S2, Table S3). The portal vertex protein has been used as marker in a number of phylogenetic studies of the T4-like phages (Zhong et al., 2002; Sullivan et al., 2008; Brewer et al., 2014). This tree placed CBB and the other Rak2-like portal proteins within a well-supported clade showing large divergence from clades representing the *Tevenvirinae* genera of *T4likevirus, Schizot4likevirus* and clades representing other unclassified T4-like phages. The result suggests that the Rak2-like phages are highly distinct from T4-like phages for which sequence data is available in the public databases (see Figure 4). The major capsid protein has also been used to conduct similar phylogenetic studies within the T4-like phages (Comeau and Krisch, 2008). A similar phylogenetic study was done with the major capsid protein of CBB (CBB_257) which also suggested the same conclusion to that obtained with the portal vertex protein (see Supplementary S2, Figure S1).

### Transcription

**Figure 5:**
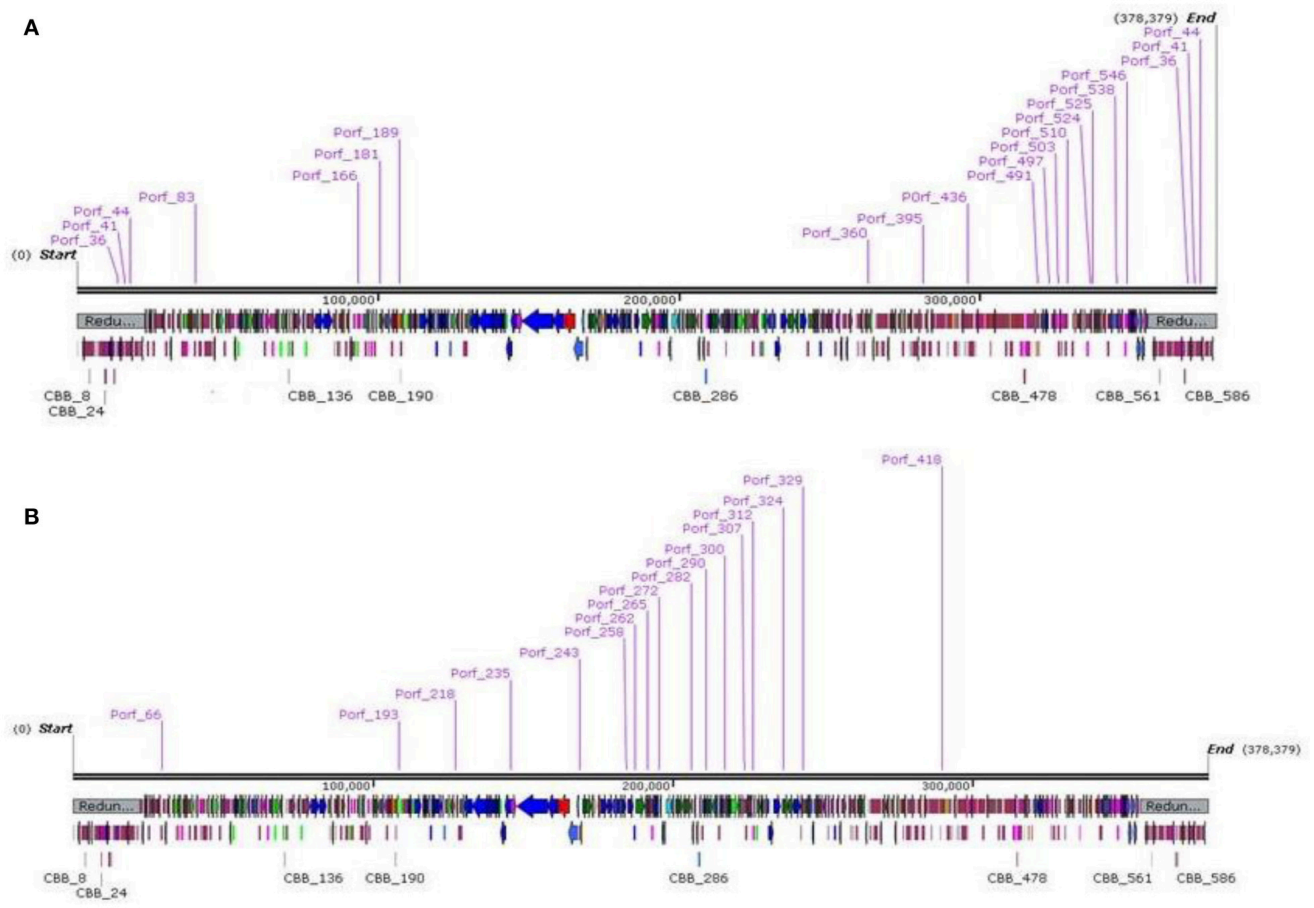
**Genome maps of bacteriophage CBB showing locations of RpoD-like promoters (A)** and CBB divergent RpoD-like promoters **(B)** created using SnapGene.

Within the genome of CBB, eighteen RpoD-like promoters were predicted with a consensus sequence preceded by an UP-like element (Estrem et al., 1998; see Supplementary S2, Table S5). Eleven of these promoters were concentrated in a region upstream of ORFs CBB_390–546, with the majority of ORFs within this region encoding short hypothetical proteins (see Figure 4 part A). Of the few ORFs for proteins of known function, two are most likely to be involved in overriding of the host transcription: the transcriptional regulator (CBB_511) and RNA polymerase sigma factor (CBB_513). The RNA polymerase sigma factor contains a RNA polymerase −70 like domain (IPR014284) containing sigma factor region 2 (IPR013325), region 3 and region 4 (IPR013324). Protein CBB_476 may also have a role in overriding host transcription due to a PrlF antitoxin family domain (IPR031848). It is suspected that the mentioned region CBB_390–CBB_546 represents the early genes which are expressed in the very early stages of infection and are involved in host take over. It should also be noted that three of the other seven RpoD-like promoters lie on the terminal repeat downstream of this region and could possibly be a continuation of the early gene region with the remaining promoters being located on the opposite end of the genome (see Figure 5 part B).

Aside from this RpoD-like promoter, a second possible promoter was detected which shows resemblance to a classic RpoD-like promoter with a classic −10 region (TATA). However, it differs with its −35 region with a consensus sequence of TGAAACG instead of TTGACA (see Supplementary S2, Table S6). This motif was also identified in the GAP32 genome (Abbasifar et al., 2014). Of the 18 examples of this detected motif, 11 were upstream of ORFs CBB_258–CBB_329 (see Figure 5 part B), with many of the ORFs within this region found to encode proteins with functions related to DNA synthesis (CBB_263, 280, 328, 290, 291, 315, 277, 324, and 325). There was also a T4-like gp55-like sigma factor (PHA02547, 4.69e-08) predicted for possible late transcription (CBB_244) just downstream of one of these promoters (Porf_244). However, no homologs for the other associated late transcription proteins of phage T4, namely gp33 and gp45, were detected (Geiduschek and Kassavetis, 2010).

A third potential promoter was found by analyzing the upstream sequences of ORFs that had been predicted to encode structure-related proteins of CBB in a single submission to MEME. Analysis showed the presence of a consensus sequence of ATAAATA with a concentration of A and T downstream of this motif. This hypothetical promoter was found to be present in 35 locations and has also been predicted to precede structural related genes in GAP32. It is expected that this sequence plays a role in late gene expression (see Supplementary S2, Table S7). This motif resembles that of the T4 late promoter sequence, namely TATAAATA (Geiduschek and Kassavetis, 2010).

Apart from the above-mentioned promoters, 74 potential rho factor independent terminators were identified in the CBB genome (Supplementary S2, Table S8).

### DNA replication, DNA modification, and nucleotide metabolism

There are eight proteins which form the T4 DNA replisome these being DNA polymerase (gp43), clamp loaders (gp44, gp62, and gp45), single strand DNA binding protein (gp32), primase (gp61), helicase (gp41), and loading protein (gp59) (Nelson et al., 2009). CBB was found to have homologs to five of the eight proteins (CBB_263, 332, 277, 290, and 291), but lacks the clamp loaders (gp62, gp45) and loading protein (gp59). Interestingly, the phage possesses a bacterial DNA polymerase III epsilon subunit (CBB_280) with a DnaQ domain (4.32e-21). There was also a protein which resembles the bacterial DNA polymerase III alpha subunit (CBB_328). However, this protein appears to be truncated, being much smaller in size to its closest bacterial hits on BLASTP (172aa vs. 1120aa) such as those from *Oceanobacillus massiliensis* (WP_010651140.1) and *B. megaterium* (WP_026681428.1).

The phage also has T4 homologs for helicase uvsw (CBB_281) and dda (CBB_315), topoisomerase gp52 (CBB_325), RNA ligase rnlA (CBB_83) and DNA ligase gp30 (CBB100), while one of its two ribonuclease H enzymes (CBB_272, 314) is related to T4 rnh. In addition, an array of T4 homologs involved in DNA repair and recombination were identified, including gp46-gp47 (CBB_246,267), endonuclease VII (CBB_248), uvsX (CBB_278), uvxW (CBB_281) and UV repair endonuclease V (CBB_519). CBB also has other DNA-replication proteins that have no counterpart to phage T4 such as primase-helicase (CBB_291) and gyrase subunit (CBB_324).

Like the other Rak2-like phages, CBB has ORFs coding for a number of enzymes apparently involved in nucleotide metabolism including the aerobic class I NrdA-NrdB (CBB_303, 305) and anaerobic class III NrdD-NrdG (CBB_141, 145) ribonucleotide reductase (RNR) enzymes for the conversion of ribonucleotides to deoxyribonucleotides (Dwivedi et al., 2013). There is also a glutaredoxin (CBB_194) to potentially support with class I RNR function (Sengupta and Holmgren, 2014). With the proteins CBB_141 and CBB_194, there are differences between phage T4 and CBB. The latter has an NrdD RNR subunit instead of the NrdH RNR subunit and glutaredoxin instead of thioredoxin. CBB has two CMP/dCMP deaminases (CBB_110,135). These generate dUMP, which is a substrate for its thymidylate synthase (CBB_223, T4 homolog td) which generates dTMP. The thymidylate synthase is supported by a dihydrofolate reductase (CBB_313, T4 homolog Frd), which provides the intermediate metabolite tetrahydrofolate. The phage has a thymidine kinase (CBB_203, T4 homolog tk) for the production of TMP, and such dNMPs may potentially be utilized by its deoxynucleoside-monophosphate kinase (CBB_203) for the production of dNDP derivatives. There is also a putative 5′, 3′ deoxyribonucleotidase (CBB_380), an enzyme in humans and mice involved in the dephosphoration of dNMP (Walldén et al., 2007).

Like GAP32, CBB has genes that appear to encode possible nicotinamide-nucleotide adenylyltransferases (CBB_113, 361) as well as a gene encoding nicotinamide phosphoribosyltransferase (CBB_115). These enzymes are involved in the *de novo* synthesis of coenzyme NAD^+^ (Schweiger et al., 2001) and their function is supported by a nicotinamide mononucleotide transporter [PnuC] (CBB_361), a membrane bound protein involved in the uptake of the NAD^+^ precursor nicotinamide mononucleotide (Zhu et al., 1991).

DNA methylation and glycosylation are strategies used by phages to provide protection against host restriction (Samson et al., 2013). CBB possesses DNA methylation enzymes for both adenine and cytosine (CBB_72, 157, 399). However, unlike GAP32, CBB lacks a second copy of DNA N-6-adenine methyltranferase (GAP32_519). No genes for enzymes were identified in the CBB genome related to the production of glycosylated hydroxymethyl cytosine which are found in the T-even phages (Petrov et al., 2010).

### tRNA gene and tRNA related protein

Phage CBB has a large number of tRNA genes and it appears that in two cases (tRNA gene 13 and 22), correct formation and function of their tRNA gene products is assisted by tyrosyl tRNA synthetase (CBB_132) and tRNAHis guanylyltransferase (CBB_152) respectively, both of which are also encoded on the CBB genome. The phage also appears to assist in tRNA turnover with the capability of releasing tRNA molecules from newly formed peptides by a tRNA peptidyl-tRNA hydrolase (CBB_392). All three gene products have homologs present in GAP32 with just CBB_132 and CBB_392 being shared with Rak2 also. As mentioned CBB has two RNA ligase enzymes (CBB_83, 181) of these CBB_83 may be involved in tRNA repair (Wang et al., 2006).

### Translation and post-translation

The phage also appears to be able to assist in the formation of the bacterial translation initiation complex (ribosome, mRNA, and tRNA) by possessing its own translation initiation factor IF-3 (CBB_318), with homologs of this protein also being identified in GAP32, 121Q, PBECO4, and Rak2. In addition, CBB has two ORFs encoding for GroES-like proteins (CBB_131,268). Large phages such as T4 and *Enterobacter* phage RB49 can use host-encoded co-chaperonin GroES to assist in the correct folding of their own structural proteins. However, these two phages also possess their own phage-encoded GroES-like proteins that can mimic bacterial host GroES proteins (Keppel et al., 2002), this may also be the case for CBB

### Terminase and DNA packing

Packaging of bacteriophage T4 DNA into its capsid requires two proteins; the small terminase (gp16) and the large terminase (gp17). Phage CBB has two proteins which possess the 17 terminase domain (PHA02533), namely CBB_274 (*E*-value 1.28e-09) and CBB_275 (*E*-value 5.47e-79). However, in the case of CBB_274 this domain is incomplete suggesting it to be a truncated form of a large terminase. This feature was also found to be present with the other Rak2-like phages and is considered to be unusual and has also been identified in the T4 superfamily *Sinorhizobium* phages phiM12 (NC_027204) and phiN3 (NC_028945).

The genomic DNA of phage T4 is packaged into its capsid with the headful packaging strategy, which results in a partially circularly permuted chromosome that is terminally repeated (Casjens and Gilcrease, 2009). The exact physical nature of the genome of CBB currently remains unknown; but as discussed previously, analysis of its genome sequence reads suggests the presence of terminal repeats.

CBB also has an ORF for a T4 homolog to endonuclease VII (CBB_248), which in T4 is involved in DNA packaging as well as recombination and mismatch repair.

### Selfish genetic elements

Homing endonucleases are selfish mobile genetic elements with endonuclease activity that only promote the spread of their own encoded gene. These can be found as self-standing genes within introns, as fusions with host proteins or also in self-splicing inteins and their presence is prevalent in a number of phage genomes (Edgell et al., 2010). In CBB, three free-standing homing endonuclease genes of the HNH family were identified, namely homing endonucleases CBB_420, CBB_ 528, and CBB_535 resembling those found in bacteriophages Rak2 and GAP32. Homing endonuclease CBB_420 lies at the end of tRNA gene 7A, CBB_528 sits between a hypothetical and a putative membrane protein and CBB_535 lies at end of virion structural protein, Inteins are selfish genetic elements which self-cleave during protein posttranslation. These elements are typically spread by homing endonuclease elements (Tori and Perler, 2011). Nevertheless, no intein-related domains (hedgehog/Hint) were found in the genome of CBB. However, it should be mentioned that inteins can occur in the Rak2-like phages with hedgehog/Hint domain (smart00306) in association with the domain of a homing endonuclease of the LAGLIDADG family (pfam14528). Both were detected in the large terminase of phages Rak2 and K64-1.

### Cell wall degrading enzymes

The 35.5 kDa gene product of CBB_187 is predicted to be the endolysin of CBB with homologs identified in GAP32 (gp180), 121Q (gp532), and RAK2 (gp506). *In silico* analysis shows that the protein is modular in structure, possessing three domains, these being an N-terminal transmembrane domain, an intermediate domain with a possible cell wall-binding domain showing weak homology to the LysM domain (IPR018392), and a C-terminus lysozyme domain (IPR023347). It is worth mentioning that the cell wall binding domain is very apparent in the 121Q homolog (PB1_121_532) of this protein. The presence of a cell binding domain is unusual in phages infecting Gram-negative bacteria and only a few endolysins with this feature have been reported to date, examples being the *Pseudomonas aeruginosa* phage endolysins KZ144 (phage φKZ), and EL188 (phage EL) (Briers et al., 2009, 2007). However, the cell-binding domains of these differ to those identified in the Rak2-like phages. Another unusual feature of this endolysin is the presence of an N-terminal transmembrane domain, reported in endolysins that follow the signal arrest system, examples being P1 lyz and φKMV gp45 lysin (Xu et al., 2004). Unsuccessful attempts to clone and express CBB_187 to date have suggested that it may be toxic, as induction results in inhibition of *E. coli* growth (data not shown).

Three identified proteins (CBB_238,239 and 240) are possibly associated with the baseplate structure of the phage tail; and each has a lysozyme domain. Gene product CBB_238 possesses a GPW 25-like domain (PF04965), which is related to gp25 of phage T4. The latter forms part of the T4 baseplate and possesses acidic lysozyme activity (Szewczyk et al., 1986), with protein CBB_238 also being quite similar in size (129 aa vs. 132 aa). Gene product CBB_239 has a T4-type lysozyme domain (IPR001165) and CBB_240 an N-terminal gp5 domain (IPR006531) with a C-terminal lysozyme (IPR002196). The gp5 N-terminal domain is an oligosaccharide-binding (OB) fold and is related to the tail spike (gp5) of T4. However, it is not certain if CBB_250 has this function as it is much larger than gp5 (894aa vs. 575aa) and the domain architectures differs, with gp5 possessing an N-terminal OB fold, centrally located lysozyme and C-terminal triple stranded-helix domains (Kanamaru et al., 2002). CBB_250 may also contain a centrally located M23 family peptidase domain (pfam01551, *E*-value 6.48E-4) which could be identified in homologs in other Rak2-like phages. Of these three proteins, only CBB_238 and CBB_240 have been confirmed as structural proteins in the Rak2-like phages.

### Structural proteins of CBB and other Rak2-like phages

**Figure 6:**
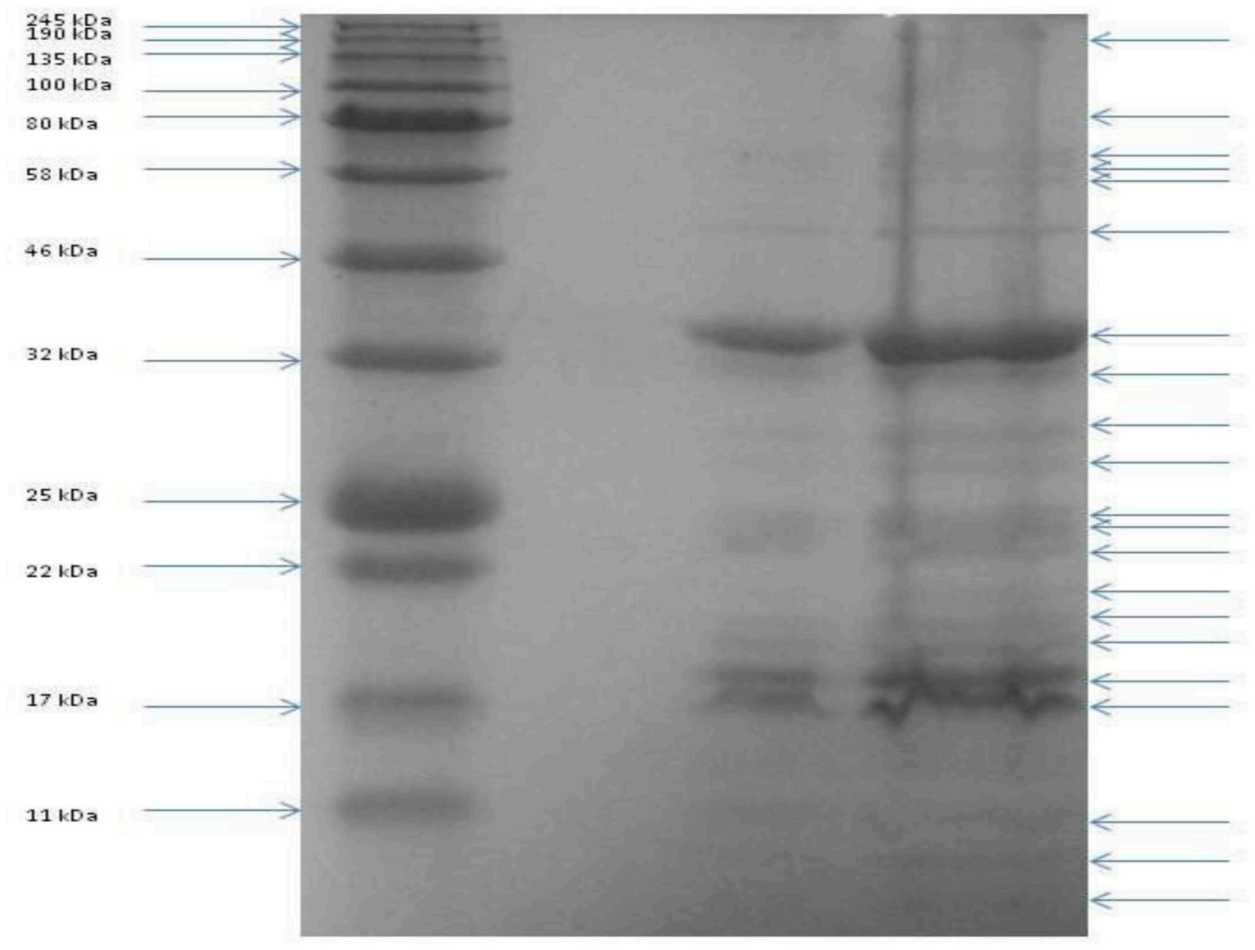
**SDS PAGE of the structural proteins of phage vB_PcaM_CBB**. Left lane shows migration patterns of New England Biolab's coloured molecular mass board range protein standard with right lanes showing that of structural proteins of phage CBB.

To identify gene products involved in the maturation and structure of the capsid of CBB, an *in silico* approach was first undertaken to identify homologs of the proteins with known functions. This was followed by identification of proteins having domains with structure-related roles and also by protein analysis using mass spectrometry (ESI-MS/MS). The identification of phage structural homologs was supported by structural proteomic analysis data that had previously been conducted on other Rak2-like phages, namely GAP32 and Rak2 (Šimolūnas et al., 2013; Abbasifar et al., 2014). Using this approach, it was possible to predict 65 gene products with structural roles (excluding the terminase proteins as well as the PhoH and ssDNA binding protein of Rak2). Such genes were identified to have functions in the structure of the capsid (CBB_208, 252, 255, 257), neck (CBB_201), tail (CBB_112, 204, 233), baseplate (CBB_237, 238, 239, 240, 242), and tail fibers (CBB_226, 260, 261). Next we attempted an experimental verification of structural proteins using SDS-PAGE of the denatured virion proteins (see Figure 6), followed by trypsin digestion and mass spectrometry. From this work, 55 proteins were associated with the structure of the phage capsid (see Table 4) with sequence coverage ranging from 4.1 to 91.3% and the number of unique peptides ranging from 1 to 30. These results allowed the identification of an additional 18 structural gene products to be associated with the structure of the mature CBB phage particle (see Supplementary S5, Table S1 and Supplementary S6).

**Table 4:** **Details of the proteins identified from ES-MS/MS conducted on capsid proteins of phage CBB**.

**ORF**	**Predicted function**	**Number of unique peptides**	**Sequence coverage %**
CBB_29	Structural protein	8	29.55
CBB_38	Structural protein	9	56.61
CBB_104	ATP-dependent Clp protease proteolytic subunit	1	4.51
CBB_112	Major tail protein	4	24.30
CBB_118	Structural protein	7	60.40
CBB_144	Structural protein	5	34.00
CBB_169	Structural protein	3	26.30
CBB_183	Structural protein	8	23.60
CBB_201	Neck protein	5	32.60
CBB_205	Structural protein	6	20.10
CBB_207	Structural protein	9	37.10
CBB_210	Structural protein	13	60.50
CBB_213	Structural protein	5	18.93
CBB_214	Structural protein	1	15.40
CBB_224	Structural protein	22	47.80
CBB_225	Structural protein	16	68.54
CBB_226	Long tail fiber proximal subunit	10	6.06
CBB_227	Structural protein	5	32.07
CBB_228	Structural protein	13	44.09
CBB_230	Structural protein	2	12.70
CBB_231	Structural protein	12	53.30
CBB_233	Tail sheath stabilizer and completion protein	12	31.61
CBB_234	Structural protein	12	35.84
CBB_236	Structural protein	21	7.72
CBB_237	Baseplate wedge	13	12.47
CBB_240	Baseplate hub subunit and tail lysozyme	3	4.11
CBB_241	Structural protein	12	17.10
CBB_242	Baseplate wedge protein	3	39.40
CBB_243	Structural protein	8	46.20
CBB_247	Structural protein	1	4.78
CBB_251	Structural protein	29	50.14
CBB_254	Structural protein	3	15.50
CBB_256	Structural protein	4	13.23
CBB_257	Precursor of major head subunit	21	68.06
CBB_260	Putative tail fiber protein	11	24.04
CBB_270	Structural protein	12	91.32
CBB_271	Structural protein	9	56.40
CBB_276	Structural protein	8	21.70
CBB_286	Structural protein	4	29.70
CBB_292	Structural protein	1	10.90
CBB_294	Structural protein	4	33.10
CBB_298	Structural protein	10	35.81
CBB_299	Structural protein	2	20.00
CBB_320	Structural protein	4	37.20
CBB_322	Structural protein	30	43.57
CBB_334	Structural protein	7	40.00
CBB_494	Structural protein	1	5.81
CBB_496	Structural protein	4	17.50
CBB_536	Structural protein	8	41.40
CBB_537	Structural protein	2	11.00
CBB_548	Structural protein	5	25.90
CBB_549	Structural protein	6	50.70
CBB_551	Structural protein	4	24.10
CBB_552	Structural protein	2	9.54
CBB_553	Structural protein	7	34.80

The structure of CBB is very similar to that of GAP32, sharing 76 homologs of the 83 gene products predicted to be involved in CBB morphology. Phage Rak2 however, is less similar to CBB, only sharing 64 homologs of these genes. TEM images taken of these phages shows that tail fibers associated with the baseplates of CBB and GAP32 are morphological very similar with both greatly differing to that of Rak2 (Šimolūnas et al., 2013; Abbasifar et al., 2014). Analysis of the structural genes also indicates this. There are tail fiber related genes (CBB_226, 261) in the genomes of GAP32 and CBB which have no homologs in Rak2 and there are also a number of tail fiber/tail spike gene products (RAK_527, 528, 530, 530, 532) in Rak2 which are not represented in CBB and GAP32. These differences most likely represent adaption of tail components correlating with host ranges of these phages.

The only structural protein of GAP32 that had previously been identified by mass spectrometry, which was not found in the genome of CBB, was GAP_001. It resembles a DNA chromosome condensation protein. This protein possesses the AST1 (COG5184) domain of the Conserved Domain Database and the regulator of chromosome condensation 1/ beta-lactamase-inhibitor protein II (IPR009091) domain of Interproscan. Interestingly homologs of this protein were also identified in multiple copies in the genomes of other Rak2-like phages 121Q (121Q_234–238, 240–242, and 244–248) and PBEC04 (ACQ_286, 289, 292, and 294–296).

The majority of the structural genes of CBB found within one large region of approximately 70 kbp (CBB_201–261), with small clusters of genes occurring throughout the rest of the genome. There are six structural proteins (CBB_144, 247, 536, 537, 551, and 552) that have been identified by mass spectrometry whose genes are not present on GAP32 genome, and four of these occur in a small ~10 kbp cluster (CBB_534–553).

One of the unusual features found with phage CBB when compared to GAP32 was a number of proteins containing Kelch-like domains for which there were no obvious homologs present in the other Rak2-like phages. These proteins were CBB_550, possessing a Kelch-type beta propeller (IPR015915) domain and CBB_552 and CBB_554, both with a Kelch-type beta propeller (IPR015915) and Kelch repeat type 1 (IPR006652) domains. These domains are comprised of six four-stranded beta propellers and have been identified as important in protein-binding interactions in a number of non-phage-related proteins. Of the three mentioned proteins, CBB_552 has been identified to form part of the mature phage structure. Proteins containing Kelch-like domains have been identified in the proteomes of eukaryotic viruses such as poxvirus. Their presence is unusual in phage but they have been reported in other giant phages that infect *Pseudomonas* and *Yersinia* (Hertveldt et al., 2005; Barry et al., 2010; Skurnik et al., 2012).

Given the high level of morphological and genetic similarity of phage CBB–GAP32, it is logical to assume that the presence of the extra morphological feature of hair-like structures along the tail shaft of phage CBB would be represented by morphological genes not present in GAP32 (other than CBB_370, a homolog of which is found in Rak2), if GAP32 does indeed lack these atypical whisker-like structures observed on CBB. However, it is still possible that a number of other structural proteins of CBB may not have been detected in the mass spectrometry assays conducted for this article. At this time, it is not possible to pinpoint the exact gene products responsible for the atypical whisker-like structures observed in phage CBB.

## Conclusion

A number of jumbo phages like CBB have been reported in recent years (Kim et al., 2013; Šimolūnas et al., 2013; Abbasifar et al., 2014; Drulis-Kawa et al., 2014; Buttimer et al., 2016; Yuan and Gao, 2016). Among them, phage CBB is significant given the identification of atypical whisker-like structures on its contractile tail, for which a function has not yet been assigned. However, given that a number of proteins found on phage particle surfaces (such as those with Ig-like domains, Fraser et al., 2006) have been proposed to play a role in the interaction between the phage and the host cell surface other than tail fiber proteins, it is not difficult to hypothesize that the whisker-like structures may play a similar role. However, this remains to be proven. The likely reason that these phages have been rarely isolated and reported may be due to the difficulty observed with plaque formation in standard plaque assay overlays, as experienced with phage CBB.

CBB is a member of a group of phages represented by phage Rak2 (the first to have been reported), of which, phages GAP32, PBEC03, 121Q, and K61-1 are members. CBB, like the other Rak2-like phages possesses a number of homologous core proteins found in T4-like phages, a number of which play crucial functions, suggesting that these proteins have not been acquired by horizontal gene transfer as seen with some phages (Petrov et al., 2010). However, given the level of divergence and the lack of a number of core T4-like protein homologs, this relationship appears to be distant with current members of *Tevenvirinae*, as suggested by the phylogenetic analysis of portal vertex and major capsid proteins done in this study. In addition, these phages also possess a large number of proteins that have not been identified in other T4-like phages, as illustrated with total protein comparison of the Rak2-like phages to those of phage T4 and KVP40.

With the sequence annotation and morphological data currently available for these phages, it is justifiable that they would form their own genus (“Rak2virus”) without any question; and indeed the proposal of placing them within a new subfamily has very strong merit, as suggested by Abbasifar et al. (2014). Nevertheless, it must be mentioned that the putative functions of the CBB genes/proteins described in this work is based on gene identification and similarity to genes/proteins of other phages in the public databases. *In vivo* work would be useful to fully validate their precise roles.

## Author contributions

CB, did the main body of research and wrote this article; HH, contributed to the mass spec work; HO, contributed to the sequencing of the phage genome; ACa, contributed to the PFG; HN, contributed to the TEMs; OM, contributed to the PFG; RR, edited article; CH, edited article; JN, contributed to the mass spec work; JM, edited article; RL, contributed to the sequencing and mass spec work; ACo edited and financed the publication.

## Funding

Funding was provided by Cork Institute of Technology as a PhD fellowship to CB.

### Conflict of interest statement

The authors declare that the research was conducted in the absence of any commercial or financial relationships that could be construed as a potential conflict of interest.
