# Supplementary material for: Things Are Getting Hairy: Enterobacteria Bacteriophage vB_PcaM_CBB
Source: Front Microbiol. 2017 Jan 24;8:44. doi: 10.3389/fmicb.2017.00044 (PMC5259590; doi:10.3389/fmicb.2017.00044)
Supplement: Supplementary file 1 [file DataSheet1.DOCX]

**Supplementary S1**

**Table S1 – Bacteria strains used in the Isolation and the testing of host range of Enterobacteria phage vb_PcaM_CBB**

| **Bacteria** | **strain** | **Isolation source** |
| --- | --- | --- |
| *Cronobacter muytjensii* | ATCC 51329 (type strain) | Unknown |
| *Cronobacter malonaticus* | DPC 6531 | Brain tumour |
| *Cronobacter sakazakii* | ATCC 29004 | Unknown |
| *Dickeya chrysanthemi bv chrysanthemi* | LMG 2804 (type strain) | *Chrysanthemum* |
| *Dickeya dianthicola* | PD 482 | *Solanum tuberosum* cv. Ostara |
| *Dickeya dianthicola* | PD 2174 | - |
| *Dickeya dianthicola* | GBBC 1538 | - |
| *Dickeya solani* | sp. PRI 2222 | - |
| *Dickeya solani* | LMG 25865 | *Solanum tuberosum* cv. Première |
| *Dickeya solani* | GBBC 1502 | - |
| *Dickeya solani* | GBBC 1586 | - |
| *Enterobacter cloacae* | NCTC 11590 | Unknown |
| *Enterobacter gergoviae* | NCTC 11434 (type strain) | Human urinary tract |
| *Erwinia amylovora* | LMG 2024 (type strain) | Pear (*Pyris communis*) |
| *Erwinia amylovora* | GBBC 403 | *Crataegus sp.* |
| *Erwinia mallotivora* | LMG 1271 | *Mallotus japonicus* |
| *Pantoea agglomerans* | LMG 2660 | *Wisteria floribunda* |
| *Pantoea agglomerans* | LMG 2570 | *Sorbus sp.* |
| *Pantoea stewartii* | LMG 2713 | *Zea mays* |
| *Pantoea stewartii* | LMG 2714 | *Zea mays* |
| *Pantoea stewartii* | LMG 2712 | *Zea mays* |
| *Pectobacterium atrosepticum* | DSM 18077 (type strain) | Potato (*Solanum tuberosum*) |
| *Pectobacterium atrosepticum* | DSM 30186 | Potato (*Solanum tuberosum* cv. Maritta) |
| *Pectobacterium atrosepticum* | CB BL5-1 | Potato (*Solanum tuberosum* cv. British Queen) |
| *Pectobacterium atrosepticum* | CB BL7-1 | Potato (*Solanum tuberosum*) |
| *Pectobacterium atrosepticum* | CB BL11-1 | Potato (*Solanum tuberosum*) |
| *Pectobacterium atrosepticum* | CB BL12-2 | Potato (*Solanum tuberosum* cv. Golden wonder) |
| *Pectobacterium atrosepticum* | CB BL13-1 | Potato (*Solanum tuberosum* cv. Golden wonder) |
| *Pectobacterium atrosepticum* | CB BL14-1 | Potato (*Solanum tuberosum* cv. Golden wonder) |
| *Pectobacterium atrosepticum* | CB BL15-1 | Potato (*Solanum tuberosum*  cv. Golden wonder) |
| *Pectobacterium atrosepticum* | CB BL16-1 | Potato (*Solanum tuberosum*  cv. Golden wonder) |
| *Pectobacterium carotovorum sbp. carovotorum* | DSM 30168 (type strain) | Potato (*Solanum tuberosum*) |
| *Pectobacterium carotovorum sbp. carovotorum* | DSM 30169 | *Brassica oleracea* var.*capitata* |
| *Pectobacterium carotovorum sbp. carovotorum* | DSM 30170 | Potato (*Solanum tuberosum* "Maritta") |
| *Pectobacterium carotovorum sbp. carovotorum* | CB BL19-1-37 | Potato (*Solanum tuberosum* cv. Golden wonder) |
