# Supplementary material for: Things Are Getting Hairy: Enterobacteria Bacteriophage vB_PcaM_CBB
Source: Front Microbiol. 2017 Jan 24;8:44. doi: 10.3389/fmicb.2017.00044 (PMC5259590; doi:10.3389/fmicb.2017.00044)
Supplement: Supplementary file 2 [file DataSheet2.DOCX]

**Supplementary S2**

**Table S1 – Proteins in the genome of phage CBB that share homology (Detected using BLASTP).**

| **Genes that share homology** |
| --- |
| CBB_47 (CBB_600) to CBB_50 (CBB_603) |
| CBB_147 to CBB_148 to CBB_149 |
| CBB_160 to CBB_161 |
| CBB_438 to CBB_439 |
| CBB_480 to CBB_481 to CBB_482 |
| CBB_536 to CBB_537 |
| CBB_539 to CBB_540 to CBB_541 to CBB_543 |
| CBB_549 to CBB_553 |
| CBB_356 to CBB_10 (CBB_563) |

**Table S2 - Quasicore proteins of the T4 like phage identified within the CBB genome with sequence comparison to homologs in Enterobacter phage T4 and Vibrio phage KVP40 (reference strains of T4likeviruses and Schizot4likeviruses, respectively) using BLASTP.**

**Conserved region of CBB portal vertex protein used to construct phylogenetic tree.**

Sequence - **DMQPHQAMAYVERVKNDIHQRRIPSNKGGSTSLMDAAYNPLSILEDYFFPQTAEGRGSSVETLPGGDNLGQIDDLRYFNNKLIRGLQIPASYLPMGPDDGGVALFGDGATQAMASELRFNNECMRYQRIISRIFDEEFKRYMIKNGYNISASSFEVTFNPPMNFAANRKAEMDAKLIQTYM**

**Table S3 – Phage portal vertex proteins used for phylogenetic study of CBB and the GAP32-like phage.**

| **Phage** | **Portal vertex protein -**  **Accession number** |
| --- | --- |
| Enterobacteria_phage_IME08 | YP_003734309.1 |
| Escherichia_phage_slur07 | YP_009197287.1 |
| uncultured_Mediterranean_phage_uvMED | BAR36277.1 |
| uncultured_Mediterranean_phage_uvMED(2) | BAR31405.1 |
| uncultured_Mediterranean_phage_uvMED(3) | BAR29473.1 |
| Synechococcus_phage_S-IOM18 | YP_008126421.1 |
| Synechococcus_phage_S-RIM2_R1_1999 | YP_007675582.1 |
| uncultured_Mediterranean_phage | ANS04762.1 |
| Enterobacteria phage RB14 | YP_002854503.1 |
| Aeromonas_phage_25 | YP_656382.1 |
| Synechococcus_phage_metaG-MbCM1 | YP_007001618.1 |
| Enterobacteria_phage_RB16 | YP_003858509.1 |
| Aeromonas_phage_65 | YP_004300925.1 |
| Aeromonas_phage_Aeh1 | NP_944108.1 |
| Aeromonas_phage_PX29 | YP_009011644.1 |
| Enterobacteria_phage_JS10 | YP_002922513.1 |
| Aeromonas_phage_Aes508 | YP_007010833.1 |
| uncultured Mediterranean phage | ANS05306.1 |
| Cyanophage_P-RSM6 | YP_007675137.1 |
| Escherichia_phage_ECML-134 | YP_009102640.1 |
| Prochlorococcus_phage_P-SSM3 | YP_008129949.1 |
| Shigella_phage_pSs-1 | YP_009110988.1 |
| Enterobacteria_phage_QL01 | YP_009202903.1 |
| Ralstonia_phage_RSP15 | BAU39992.1 |
| uncultured_Mediterranean_phage | ANS05306.1 |
| uncultured_Mediterranean_phage(2) | ANS05306.1 |
| uncultured_Mediterranean_phage_uvMED | BAR34134.1 |
| Cyanophage_P-RSM1 | YP_007877738.1 |
| Cyanophage_Syn30 | YP_007877943.1 |
| Escherichia_phage_121Q | YP_009102185.1 |
| Synechococcus_phage_S-CAM8 | YP_008125640.1 |
| Synechococcus_phage_S-RIM8_A.HR1 | YP_007518198.1 |
| Synechococcus_phage_syn9 | YP_717798.1 |
| Synechococcus_phage_S-CRM01 | YP_004508471.1 |
| Synechococcus_phage_S-RSM4 | YP_003097343.1 |
| Cyanophage_S-RIM50 | AMO42907.1 |
| Enterobacteria_phage_RB27 | YP_009102372.1 |
| Enterobacteria_phage_RB3 | YP_009102372.1 |
| Enterobacteria_phage_RB32 | YP_803110.1 |
| Escherichia_phage_vB_EcoM-UFV13 | ANA50202.1 |
| Salmonella_phage_STML-198 | YP_009148150.1 |
| Shigella_phage_SHFML-11 | ANN86599.1 |
| Aeromonas_phage_44RR2.8t | NP_932511.1 |
| Cyanophage_P-TIM40 | YP_009188207.1 |
| Cyanophage_S-RIM32 | AMO43137.1 |
| Enterobacteria_phage_RB69 | NP_861872.1 |
| Enterobacteria_phage_vB_EcoM_VR20 | YP_009207360.1 |
| Enterobacteria_phage_vB_EcoM_VR5 | YP_009205862.1 |
| Enterobacteria_phage_vB_KleM-RaK2 | YP_007007244.1 |
| Escherichia_phage_APCEc01 | YP_009225085.1 |
| Escherichia_phage_PBECO_4 | SCA80472.1 |
| Escherichia_phage_vB_EcoM_JS09 | YP_009030623.1 |
| Aeromonas_phage_CC2 | YP_007010339.1 |
| Cronobacter_phage_vB_CsaM_GAP32 | YP_006987350.1 |
| Edwardsiella_phage_PEi20 | YP_009190346.1 |
| Enterobacteria_phage_Bp7 | YP_007004276.1 |
| Enterobacteria_phage_JSE | YP_002922232.1 |
| Escherichia_phage_e11/2 | YP_009030776.1 |
| Escherichia_phage_UFV-AREG1 | ANH50304.1 |
| Escherichia_phage_vB_EcoM_PhAPEC2 | YP_009056756.1 |
| Pelagibacter_phage_HTVC008M | YP_007517949.1 |
| Prochlorococcus_phage_P-HM1 | YP_004322541.1 |
| Prochlorococcus_phage_P-HM2 | YP_004323487.1 |
| Prochlorococcus_phage_P-RSM4 | YP_004323264.1 |
| Prochlorococcus_phage_P-SSM2 | YP_214363.1 |
| Prochlorococcus_phage_P-SSM4 | YP_214665.1 |
| Prochlorococcus_phage_P-SSM7 | YP_004324951.1 |
| Prochlorococcus_phage_Syn33 | YP_004323727.1 |
| Sinorhizobium_phage_phiM12 | YP_009142980.1 |
| Sinorhizobium_phage_phiN3 | YP_009212309.1 |
| Synechococcus_phage_S-PM2 | YP_195138.1 |
| Synechococcus_phage_S-ShM2 | YP_004322786.1 |
| Synechococcus_phage_S-SM1 | YP_004323020.1 |
| Synechococcus_phage_S-SSM5 | YP_004324725.1 |
| Synechococcus_phage_S-SSM7 | YP_004324197.1 |
| Vibrio_phage_KVP40 | BAA77377.1 |
| Vibrio_phage_nt-1 | YP_008125180.1 |
| Yersinia_phage_PST | YP_009153767.1 |
| Prochlorococcus_phage_MED4-213 | YP_007673752.1 |
| Salmonella_phage_S16 | YP_007501199.1 |
| Salmonella_phage_vB_SnwM_CGG4-1 | ANA49508.1 |
| Synechococcus_phage_ACG-2014a | AIX28031.1 |
| Synechococcus_phage_ACG-2014b | YP_009140680.1 |
| Synechococcus_phage_ACG-2014d | AIX21202.1 |
| Synechococcus_phage_ACG-2014e | YP_009134611.1 |
| Synechococcus_phage_ACG-2014f | AIX42351.1 |
| Synechococcus_phage_ACG-2014g | YP_009133666.1 |
| Synechococcus_phage_ACG-2014h | YP_009008243.1 |
| Synechococcus_phage_ACG-2014i | YP_009140894.1 |
| Synechococcus_phage_ACG-2014j | YP_009134101.1 |
| Synechococcus_phage_S-MbCM100 | YP_009007965.1 |
| Shigella_phage_Shfl2 | YP_004415062.1 |
| Escherichia_phage_HY03 | AKJ72700.1 |
| Vibrio_phage_VH7D | YP_009006286.1 |
| uncultured_phage_MedDCM-OCT-S09-C7 | ADD95604.1 |
| Aeromonas_phage_phiAS4 | YP_003969129.1 |
| Aeromonas_phage_phiAS5 | YP_003969303.1 |
| Klebsiella phage vB_KpnM_KpV477 | ANT40618.1 |
| Klebsiella phage JD18 | YP_009190743.1 |
| Enterobacteria phage T4 | NP_049782.1 |

**Figure S1 -** Maximum likelihood tree created from the alignment of the major capsid protein of 100 homologous sequences from different T4-like phages to that of major capsid protein of phage CBB found using a BLASTP search.

**
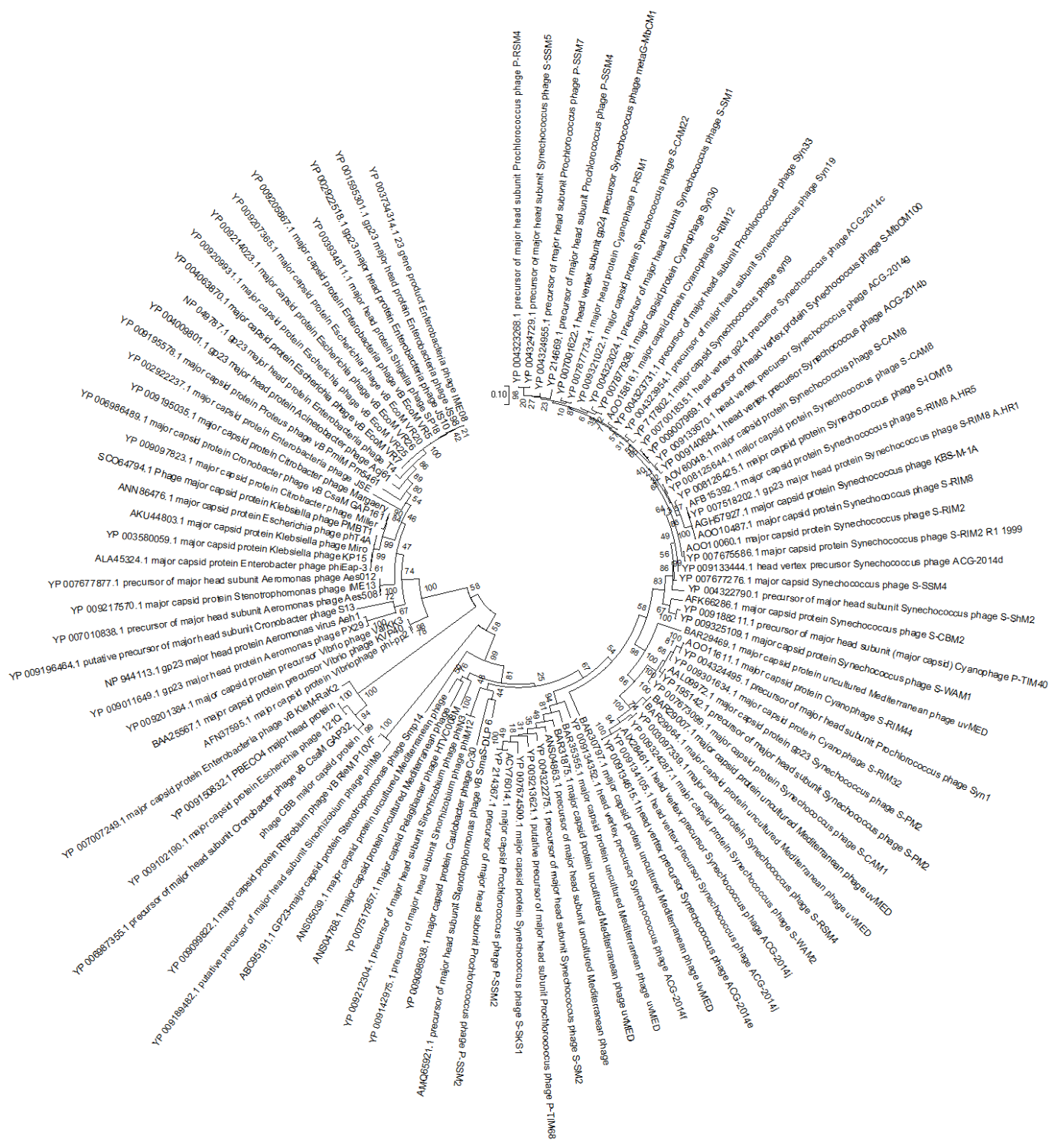
**

**Table S4 – tRNA gene in the genome of phage CBB (Detected using tRNAscan –SE and ARAGORN).**

| Feature | Location | Function | Predection program |
| --- | --- | --- | --- |
| tRNA gene 1 | 268,792 bp - 268,878 bp | Ser, anticodon GCT | tRNAscan-SE |
| tRNA gene 1A | 272,675 bp - 272,749 bp | tRNA-Trp(cca) | ARAGORN |
| tRNA gene 1B | 275,819 bp - 275,892 bp | tRNA-Thr(tgt) | ARAGORN |
| tRNA gene 2 | 276,549 bp - 276,669 bp | Val, anticodon AAC (intron present 276,585 bp – 276,634 bp) | tRNAscan-SE |
| tRNA gene 3 | 280,358 bp - 280,440 bp | Leu, anticodon TAG | tRNAscan-SE |
| tRNA gene 4 | 288,346 bp – 288,417 bp | Arg, anticodon TCT | tRNAscan-SE |
| tRNA gene 5 | 289,995 bp -290,067 bp | Sup, anticodon CTA or tRNA-Pyl(cta) | TRNAscan -SE ARAGORN |
| tRNA gene 6 | 290,284 bp – 290,367 bp | Leu, anticodon TAA | tRNAscan-SE |
| tRNA gene 7 | 290,373 bp – 290,482 bp | Val, anticodon GAC (intron present 290,373 bp – 290,482 bp) | tRNAscan-SE |
| tRNA gene 7A | 290,748 bp -290,824 bp | tRNA-Met(cat) | ARAGORN |
| tRNA gene 8 | 292,673 bp -292,754 bp | Leu, anticodon TAA | tRNAscan-SE |
| tRNA gene 9 | 295,724 bp – 295,837 bp | Pseudo tRNA | tRNAscan-SE |
| tRNA gene 10 | 297,425 bp -297,546 bp | Phe, anticodon GAA, (intron present 297,459 bp – 297,510 bp) | tRNAscan-SE |
| tRNA gene 11 | 299,090 bp -299,176 bp | Leu, anticodon CAA | tRNAscan-SE |
| tRNA gene 12 | 302,062 bp – 302,132 bp | Glu, anticodon TTC | tRNAscan-SE |
| tRNA gene 13 | 302,143 bp -302,231 bp | tRNA-Tyr(gta) | ARAGORN |
| tRNA gene 14 | 302,564 bp - 302,654 bp | tRNA-Ser(tga) | ARAGORN |
| tRNA gene 15 | 302,662 bp - 302,780 bp | Ser, anticodon AGA (intron present 302,696 bp – 302,744 bp) | tRNAscan-SE |
| tRNA gene 16 | 304, 228 bp – 304,298 bp | Ile, anticodon GAT | tRNAscan-SE |
| tRNA gene 16A | 307,123 bp -307,195 bp | tRNA-Asn(gtt) | ARAGORN |
| tRNA gene 17 | 307,717 bp – 307,789 bp | Gln, anticodon TTG | tRNAscan-SE |
| tRNA gene 17A | 308,195 bp - 308,267 bp | tRNA-Gly(tcc) | ARAGORN |
| tRNA gene 18 | 309,726 bp -309,787 bp | Asp, anticodon GTC | tRNAscan-SE |
| tRNA gene 19 | 310, 643 bp – 310,718 bp | Arg, anticodon ACG | tRNAscan-SE |
| tRNA gene 20 | 311,782 bp – 311,853 bp | Pro, anticodon TGG | tRNAscan-SE |
| tRNA gene 21 | 311,859 bp - 311,931 bp | Pro, anticodon TGG | tRNAscan-SE |
|  |  |  |  |
| tRNA gene 22 | 312,275 bp – 312,348 bp | His, anticodon GTG | tRNAscan-SE |
| tRNA gene 23 | 312, 637 bp – 312,710 bp | Phe, anticodon GAA | tRNAscan-SE |
| tRNA gene 24 | 313,823 bp – 313,895 bp | Lys, anticodon TTT | tRNAscan-SE |
| tRNA gene 25 | 318,802 bp – 318,876 bp | Cys, anticodon GCA | tRNAscan-SE |
| tRNA gene 26 | 319,754 bp – 319,826 bp | Met, anticodon CAT | tRNAscan-SE |
| tRNA gene 26A | 320,334 bp-320,411 bp | tRNA-Met(cat) | ARAGORN |
| tRNA gene 27 | 320, 479 bp – 320,511 bp | Ala, anticondon TGC | tRNAscan-SE |

**Table S5 - Promoter 2 of Phage CBB found using MEME**


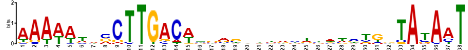


| Promoter | location | **Sequence** |
| --- | --- | --- |
| Porf_36 | 13,365-13,402 | AAAAATAGCTTGCATCACTCGTAATTATGAAGTATAAT |
| Porf_41 | 15,759-15,796 | AAAAAGGAGTTGACAACAGTTGGCTCCTTTGCTATACT |
| Porf_44 | 17,515-17,552 | AAAAATTCCCTTGCTTTCTTTCTTTCGTGTGGTATACT |
| Porf_83 | 39,070-39,107 | AATACTGACTTGACACGAGGCAGCAAGAGTGTTACAAT |
| Porf_166 | 93,493-93,530 | AAAAATAGCTTGATTTTGAGCTACATAATTGATATAAT |
| Porf_181 | 100,667`-100,704 | ACAAATTGCTTGACATGGCAGTGAGTATCATATATCAT |
| Porf_189 | 106,894-106,931 | GAATAAACCTTGACAGTACGATAAAAAAGTGCTACATT |
| Porf_360 | 262,781-262,818 | AAATTTACCTTGACAAAATCAACTCCATCCCTGATAAT |
| Porf_395 | 281,079-281,116 | AAAAATGACTTGGCAGGAGATTAATATTCCTGTACAAT |
| Porf_436 | 296,151-296,188 | TAAAAATCCTTGACTTGCGTCGGTTGACCTGATAGAAT |
| Porf_491 | 319,303-319,340 | CAAAACACCTTGACAGTGGCATCCTCTCATGGCAAAAT |
| Porf_497 | 323,285-323,322 | AGATTTTGCTTGACAAGACGGCATTTTTTGTTTATAAT |
| Porf_503 | 326,287-326,324 | AAATACCAGTTGACTCAACGTGGGTTAACTGGTATACT |
| Porf_510 | 329,125-329,162 | AATTTGGCCTTGCAATCAGCGAACGCATGTGATATATT |
| Porf_524 | 336,865-336,902 | AGAAAATCCTTGACTTGCTTCAGCAAGGCGAGTACACT |
| Porf_525 | 337,752-337,789 | AAAAATACATTGACGTGGCACATTTTTTGTGCCATAAT |
| Porf_538 | 345,724-345,761 | TATTTAGGCTTGACACGATGTTAAATCTTTGATATAAT |
| Porf_546 | 349,130-349,167 | TAATTTGGCTTGACTTAGGCTTTCGAACTCCGTATAAT |

**Table S6 – Promoter 1 of Phage CBB found using MEME**


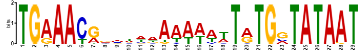


| Promoter | Location | Sequence |
| --- | --- | --- |
| Porf_66 | 29,539-29,567 | TGAAACAGAATAATTTTTTATGGTATAAT |
| Porf_113 | 54,897-54,925 | TGAAACGTAAGGAATTTTTCTGCTATAAT |
| Porf_193 | 108,832-108,860 | TGAAACAGCTTTAAAAATTATGGTATAGT |
| Porf_218 | 127,475-127,503 | TGAAACGTACCAAAATTTTATGGTATAAT |
| Porf_235 | 145,858-145,886 | TGGAACGACCTAAAAAAATGTGCTATAAT |
| Porf_244 | 168,994-169,022 | TGGAACGTTTTTTAAAATTGTGGTATAAT |
| Porf_258 | 184,600-184,628 | TGAAACGTATACATTTATTATGGTATAAT |
| Porf_262 | 187,472-187,500 | TGAAACGAAAATAAAACGTATGATATAAT |
| Porf_265 | 191,483-191,511 | TGAAACGGGTACATTTTTTATGATATAAT |
| Porf_272 | 195,587-195,615 | TGAAACTGCACATAAAAATGTGGTATAAT |
| Porf_282 | 206,162-206,190 | TGGAATGAGTTAAATTTTTGTGATATAAT |
| Porf_290 | 211,052-211,080 | TGGAATGCATTAAAAAAATGTGGTATAAT |
| Porf_300 | 217,526-217,554 | TGAAACAATAAAAAAACATGTGCTATAAT |
| Porf_307 | 224,015-224,043 | TGAAACGCCAAAATAAAATATGGTATAAT |
| Porf_312 | 226,886-226,914 | TGAAACAGCTTGGAATATTATGATATAAT |
| Porf_324 | 236,752-236,780 | TGAAACAAAGAAACAAATTATGCTATAAT |
| Porf_329 | 243,482-243,510 | TGAAATGGTAAAAAAAATTATGGTATAAT |
| Porf_418 | 289,731-289,759 | TGAAACGGCGATAAAAACTGTGGTATAAT |

| Promoter | Location | Sequence |
| --- | --- | --- |

**Table S7- Promoter 3 of Phage CBB found using MEME based on analysis of 100bps upstream regions of structural genes**


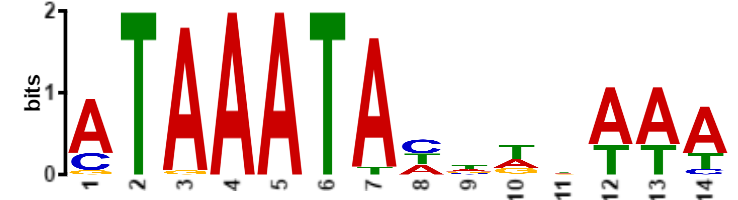


| Promoter | Location | Sequence |
| --- | --- | --- |
| Porf_112 | 54,769-54,782 | ATAAATATTAATAT |
| Porf_165 | 93,137-93,150 | ATGAATACCGCAAA |
| Porf_169 | 95,252-95,265 | CTAAATACGGTATC |
| Porf_201 | 111,914-111,927 | CTAAATATGATATA |
| Porf_204 | 133,744-113,757 | ATAAATAACATTAT |
| Porf_205 | 116,515-116528 | ATAAATACATTAAA |
| Porf_206 | 177,288-117,301 | CTAAATATAATATA |
| Porf_210 | 120,228-120,241 | ATAAATAAAATAAA |
| Porf_213 | 122,794-122,807 | CTAAATTCATTAAA |
| Porf_224 | 132,558-132,571 | ATAAATACTGGATA |
| Porf_226 | 137,986-137,999 | ATAAATACTTGAAA |
| Porf_227 | 138,970-138,983 | ATAAATATAAGAAA |
| Porf_228 | 140,195-140,208 | ATAAATATTTGTAA |
| Porf_229 | 140,234-140,247 | ATAAATAAGTAAAA |
| Porf_234 | 145,841-145,854 | ATAAATACGGTATA |
| Porf_236 | 158,306-158,319 | ATAAATAAAACAAA |
| Porf_237 | 161,845-161,858 | ATAAATATTTCTAT |
| Porf_239 | 162,810-162,823 | ATAAATACTTATAC |
| Porf_243 | 168,976-168,989 | ATAAATACTTGATA |
| Porf_250 | 176,224-176,237 | ATAAATAACTAATA |
| Porf_251 | 176,198-176211 | ATAAATACGACTAT |
| Porf_256 | 182,056-182,069 | ATAAATATCTTTAC |
| Porf_260 | 185,404-185,417 | ATAAATATTTCTAT |
| Porf_271 | 195,498-195,511 | ATAAATAACATTAA |
| Porf_276 | 199,519-199,532 | ATAAATATATCTAT |
| Porf_289 | 210,031-210,044 | ATAAATATTGTATA |
| Porf_298 | 216,496-216,509 | CTAAATAAGGATAT |
| Porf_302 | 218,818-218,831 | CTAAATAATTCTAT |
| Porf_320 | 231,630-231,643 | ATAAATACTCAAAA |
| Porf_496 | 323,194-323,207 | ATAAATACTTTATA |
| Porf_534 | 343,023-343,036 | ATAAATAAATGAAA |
| Porf_537 | 345,717-345,730 | CTAAATAATTAATA |
| Porf_551 | 353,119-353,132 | CTAAATTTTACAAA |
| Porf_552 | 353,975-353,988 | GTAAATACATAATA |
| Porf_553 | 354,727-354,740 | GTAAATACAGGATA |

**Table S8- Terminators of Phage CBB detected using ARNold and confirmed using Mfold Quikfold**

| Terminator | Coordinates | Sequence | ΔG kcal/mol |
| --- | --- | --- | --- |
| Torf8 | 3,939-3,974 | GGGGGAACATGAAAGTTCCCCTgTTTTTTTCCTTGA | -13.8 |
| Torf18 | 7,952 - 7,980 | GGGGAACATTGTTCCCCTTTTTAAGTGAG | -10.9 |
| Torf26 | 10,171 - 10,208 | AGTACTGGTGAAATATCCAGTACTgTTTTTATGTACAG | -11.4 |
| Torf39 | 15,175 - 15,205 | AGGTTGACTTCGGTCGGCCTtTTTTGTTATACTT | -11.8 |
| Torf47 | 19,234 - 19,268 | GGGCAACCAAATAACGGTTGCCCTTTTTTGTATTC | -15.3 |
| Torf52 | 22,047 - 22, 081 | GCCGCTGTCATAATGACGGCGGCTTACTTTATGGG | -18 |
| Torf67 | 30,374 - 30,401 | GGGGCTTAATTGCCCCTTTTCTATAGGA | -11.1 |
| Torf70 | 32,322 - 32, 354 | GGACGGTACAGTGTATCGTCCTTTTTTAATTGA | -12.2 |
| Torf80 | 38,447 - 38,475 | GCCCCGAAATTCGGGGCTTTCTTTTTAAA | -13.4 |
| Torf87 | 41,945 - 41,972 | GGGATACTTCGGTATCCCTTCTTTTTTAAT | -14 |
| Torf106 | 51,742- 51,774 | AGGCCACTTAGTTGTGGCCTTTTTCTTTTAAGG | -12.6 |
| Torf111 | 54,040 - 54,067 | GGAGCCATATGGCTCCTTTTTTATTTTA | -11.9 |
| Torf113 | 56,086 - 56, 116 | GGAGTACTTCGGTACTCCaTTTTAAGTAAGA | -14.4 |
| Torf123 | 63,856 - 63, 887 | GGGGATACTTAGGTATCCCCTTTTTAATTGGA | -14.3 |
| Torf124 | 64,214 - 64,242 | GGCCACGCAATGTGGCCTTTTTTATTGGA | -11 |
| Torf146 | 76-523 - 76,553 | AGCACCTGCAAGGGTGCTTTTTTTGTTTATA | -10 |
| Torf149 | 84,839 - 84, 869 | AGCGGTCACTTGACCGCTTTTTTCTTTTGTG | -11.6 |
| Torf152 | 86,569 - 86,596 | GGGAGTGTTAGCTCCCTTTTACATTTCA | -10 |
| Torf163 | 92,758 - 92, 787 | CCCGTCATAAATGGCGGGTTTTCTATTAAA | -10.4 |
| Torf165 | 93,533 - 93,565 | AGGAAATCGTGAGATTTCCTTTTTTCGTTTGGA | -12.3 |
| Torf173 | 97,558 - 97,590 | TGGGCTGATATAATCAGTCCATATTTTTATGGG | -11 |
| Torf183 | 104,263 - 104, 292 | GCCTCACTCCGGTGGGGCTTTTTCATTTAA | -12 |
| Torf188 | 106,936 - 106, 971 | GGACAGGCAGCGATGCTCTGTCCTTTTTTATTTTAA | -13.8 |
| Torf197 | 110,637 - 110, 666 | GGGCATCTTAGGATGCCCTTTTTTCAATTG | -13 |
| Torf202 | 113,016 - 113,052 | GGAAGAATCTTAATAGGGTTCTTCCTTTTTTCGTTTA | -13.2 |
| Torf204 | 116,418 - 116,485 | GGAGCCATATGGCTCCTTTTTTGTTTTC | -11.9 |
| Torf205 | 117,258 - 117, 289 | GCCCTGGACTTGTCTAGGGCTTTTTTATTGCT | -14.7 |
| Torf215 | 124,614 - 124, 647 | AGCACTGAAAACTCAGTGCTtTTCTTTTTATCAA | -10.9 |
| Torf216 | 126, 042 - 126, 072 | AGGGGCATTATGCCCCTtTTGTTTTATGAGG | -11.2 |
| Torf224 | 130,573 - 130,603 (complement) | AGGGAGCTTTTGCTCCCTTTCTTTTTACACC | -12.6 |
| Torf223 | 130,586 - 130, 616 | AGGGAGCAAAAGCTCCCTTTTTTTGTTATTG | -12.1 |
| Torf225 | 132,578 - 132, 611 | AGGTCAACTTATGTTGGCCTtTTGTTTTTTGTAG | -10.6 |
| Torf228 | 138, 985 - 139, 017 (complement) | AGGGAGATACTTATCTCCCTTTTTTCATTGTGA | -11.2 |
| Torf236 | 148, 214 - 148, 255 (complement) | GGGAGCCATATGGCTCCCTTTTTCTTTATTT | -15.2 |
| Torf235 | 148,227 - 148, 256 | GGGAGCCATATGGCTCCCTTTTTTGTTATA | -15.2 |
| Torf237 | 158,318 - 158, 348 (complement) | GGGCATCTTCGGATGCCCTTTATTCTTGCAT | -16 |
| Torf246 | 173, 110 - 173, 140 | AGGAGCCATTAGGCTCCTTTTTTTATGATAT | 12.1 |
| Torf247 | 173, 095 - 173, 128 (complement) | AAGGAGCCTAATGGCTCCTTtTTGTTTATAGGTC | 13.5 |
| Torf257 | 184, 538 - 184, 566 | GGACGCTTTATGCGTCCTTTTCGCATTTC | -11.3 |
| Torf261 | 187,504 - 187, 543 | GGCATCCTAGCGGAGAGTTAGGATGCTTTTTTTATACATG | -17.1 |
| Torf262 | 187,863 - 187, 892 | TGCCCCAGGATTGGGGCGTTTTGTTTTAAG | -11.6 |
| Torf265 | 192,281 - 192, 313 | AGGGAGCGTAATGCTCCCTcTTTTTATTTGGAT | -11.7 |
| Torf269 | 194, 180 - 194, 209 | GGAGAACATATGTTCTCCTTTTTCTTTTTT | -10.7 |
| Torf270 | 174, 166 - 194, 198 (complement) | AGGAGAACATATGTTCTCCTTTTTTTATTACTC | -11.2 |
| Torf277 | 202, 130 - 202, 164 | TTGGGGCAACGGATTTGCCCCGATTTCATTTCATG | -13.7 |
| Torf279 | 203, 777 - 203, 808 | AGGACGCTTATTGCGTCCTTTTTGTTTTTTAG | -11.8 |
| Torf303 | 221, 845 - 221, 873 | TCCCGTCTATGACGGGATTTTTTATCAGG | -10.4 |
| Torf305 | 223,790 - 223, 816 | GGAGGCTTTGCCTCCTTTTTCATGGAT | -10 |
| Torf310 | 225,406 - 225, 434 (complement) | GGGAGCTTACGCTCCCTTTTTTATGCATT | -11.5 |
| Torf323 | 236,722 - 236, 757 | TAACGACGCTCCAGAGCGTCGTTATTTTTTTGAAAC | -15 |
| Torf325 | 240,252 - 240,279 | GGACGCATAAAGCGTCCTTTATTTTTAAT | -10.8 |
| Torf330 | 244,250 - 244, 279 | GCCCTGCATTGTCAGGGCTTTCTTTTTGTC | -10.5 |
| Torf333 | 246,154 - 246, 182 | AGGGGAGTAATCCCCTTTTCCGTTTAGTA | -11 |
| Torf338 | 249,187 - 249, 219 | TGGGGAATGAAAGTTCCCCGcTTTTTAATTTAA | -13.5 |
| Torf340 | 251, 689 - 251, 720 | AGCTCCCAACACTTGGGGGCTTTTTTCATATG | -12.1 |
| Torf344 | 254, 455 - 254, 489 | AGCTGGCCTTAGTTGGTCAGCTTTTTTAAGTAAGA | -13.2 |
| Torf364 | 265, 682 - 265, 712 | CCCTGTACTAAGTACAGGGTTTTTAATTGAG | -11.4 |
| Torf446 | 300, 688 - 300, 723 | TGCCGCCAACATTGTTGGCGGTAcTTTTAAGTTAGA | -15.4 |
| Torf448 | 301, 467 - 301, 490 | GGGCGAAAGCCCTTTTTTAATTGA | -10.8 |
| Torf468 | 311, 288 - 311, 323 | GGGATTTTCCGTAAGGGAAATCCCTTTTTTCGTTTA | -17.8 |
| Torf488 | 318, 141 - 318, 173 | AGGGAGCGGATTGCTCCCTgTTTTATAATGCAT | -12.3 |
| Torf494 | 321, 450 - 321, 478 | GGCAGCCATTGGCTGCCTTTTTTCGTTTA | -13.1 |
| Torf495 | 322, 016 - 322, 046 | GGCACTCATAAAGAGTGCCTTTTGTTTTACG | -12.6 |
| Torf496 | 322,001 - 322, 035 (complement) | AGGCACTCTTTATGAGTGCCTtTTTTTCTTGTATA | -13.6 |
| Torf500 | 325, 314 - 325, 350 | TTCGGGGATTAACTATCCCCGAAacTTTTAATGGTGA | -14.6 |
| Torf515 | 333, 025 - 333, 050 | GGGTTGACTTCGGTCAGCCCTTTATTTTAATT | -16.8 |
| Torf518 | 334,640 - 334, 671 | GGGTTGACTTCGGTCAGCCCTTTTTTGTATAA | -16.8 |
| Torf520 | 335, 395 - 335, 426 | GGGTTGACTTCGGTCAGCCCTTTTTGCTATAC | -16.8 |
| Torf525 | 338, 856 - 338, 888 | TGCCAAACATCGTGTTTGGCATTTTTTATTAAA | -11.2 |
| Torf542 | 348,061 - 348, 089 | ACTCCCTACGGGGAGTTTTTTATTGAGGC | -11.6 |
| Torf546 | 349, 505 - 349, 535 | CCGCCAGCATTGTTGGCGGTTTTTTAATTGC | -12.5 |
| Torf547 | 350, 176 - 350, 206 | AAGCCCCAAAAGGGGCTTTTTTAATTTGTGA | -11.8 |
| Torf548 | 350,166 - 350, 191 | GCCCCAAAAGGGGCTTTTTTAATTTG | -10.4 |
| Torf553 | 353, 992 - 354, 024 (complement) | AGCTCAACTTAGGTTGGGCTTTTTCTGTTTTTG | -10.1 |
